# Genome-Wide Identification and Characterization of Four Gene Families Putatively Involved in Cadmium Uptake, Translocation and Sequestration in Mulberry

**DOI:** 10.3389/fpls.2018.00879

**Published:** 2018-06-29

**Authors:** Wei Fan, Changying Liu, Boning Cao, Meiling Qin, Dingpei Long, Zhonghuai Xiang, Aichun Zhao

**Affiliations:** State Key Laboratory of Silkworm Genome Biology, Key Laboratory of Sericultural Biology and Genetic Breeding, Ministry of Agriculture, Southwest University, Chongqing, China

**Keywords:** cadmium, *Morus*, transporter genes, bioinformatics analysis, phytoremediation

## Abstract

The zinc-regulated transporters, iron-regulated transporter-like proteins (ZIPs), the natural resistance and macrophage proteins (NRAMP), the heavy metal ATPases (HMAs) and the metal tolerance or transporter proteins (MTPs) families are involved in cadmium (Cd) uptake, translocation and sequestration in plants. Mulberry (*Morus* L.), one of the most ecologically and economically important (as a food plant for silkworm production) genera of perennial trees, exhibits excellent potential for remediating Cd-contaminated soils. However, there is no detailed information about the genes involved in Cd^2+^ transport in mulberry. In this study, we identified 31 genes based on a genome-wide analysis of the *Morus notabilis* genome database. According to bioinformatics analysis, the four transporter gene families in *Morus* were distributed in each group of the phylogenetic tree, and the gene exon/intron structure and protein motif structure were similar among members of the same group. Subcellular localization software predicted that these transporters were mainly distributed in the plasma membrane and the vacuolar membrane, with members of the same group exhibiting similar subcellular locations. Most of the gene promoters contained abiotic stress-related *cis*-elements. The expression patterns of these genes in different organs were determined, and the patterns identified, allowing the categorization of these genes into four groups. Under low or high-Cd^2+^ concentrations (30 μM or 100 μM, respectively), the transcriptional regulation of the 31 genes in root, stem and leaf tissues of *M. alba* seedlings differed with regard to tissue and time of peak expression. Heterologous expression of *MaNRAMP1, MaHMA3, MaZIP4*, and *MaIRT1* in *Saccharomyces cerevisiae* increased the sensitivity of yeast to Cd, suggested that these transporters had Cd transport activity. Subcellular localization experiment showed that the four transporters were localized to the plasma membrane of yeast and tobacco. These results provide the basis for further understanding of the Cd tolerance mechanism in *Morus*, which can be exploited in Cd phytoremediation.

## Introduction

Due to increasing industrial demands, global cadmium (Cd) extraction from mining increased overall by 19.1% between 2000 and 2016. In 2016, mine production of recoverable Cd was 23000 tons, with China and Korea being the major producers of Cd, with 7400 and 4500 tons of Cd production, respectively (USGS, 2017). Cd is one of the most toxic non-essential elements to organisms. Chronic human exposure to Cd at high concentrations (>30 μg⋅d^-1^) has the potential to cause kidney damage, bone lesions (itai–itai disease), cancer and lung insufficiency (Figueroa, 2008; Gad, 2014). The main route of human exposure to Cd is through the diet, due to soil Cd pollution (Clemens et al., 2013). A number of Cd-contaminated soil remediation techniques have been researched; compared with many physical and chemical approaches to this problem, phytoremediation is a more environmentally friendly and cost-effective method (Raskin et al., 1997).

In previous studies on phytoremediation, most of them focused on the identification of Cd hyperaccumulator species, such as *Arabidopsis halleri, Sedum alfredii*, and *Noccaea* (syn.*Thlaspi*) *caerulescens*, the aerial parts of which can accumulate Cd to >100 μg⋅g^-1^ (Kramer, 2010; Cun et al., 2014). However, most hyperaccumulators are herbaceous plants which have limitations, such as metal selectivity, low biomass, shallow root systems and slow growth rates. Therefore, some fast-growing woody plants have been recommended recently as potential candidates for phytoremediation (Luo et al., 2016). Mulberry exhibit desirable traits such as being highly adaptable, perennial, capable of withstanding pruning, with a high biomass and a widespread distribution (Olson and Fletcher, 2000; Zhao et al., 2013). Mulberry has been shown to be tolerant of Cd, Cr, Pb, Co, and Cu (Ashfaq et al., 2012; Zhao et al., 2013; Zhou et al., 2015a,b). After growing in soil containing 60 μg⋅g^-1^ Cd for 4 months, the Cd concentration in roots of *Morus alba* was 95.62 μg⋅g^-1^ (accumulating Cd to concentrations greater than present in the soil), while in leaves it was 6.48 μg⋅g^-1^ (Zhou et al., 2015a). Although *M. alba* is not a Cd hyperaccumulator, it can still accumulate Cd on a large scale because of its large biomass. According to Zhou et al. (2015a), in the presence of increasing Cd concentrations in mulberry leaves, detoxification mechanisms of the silkworm, feeding on its food plant, were activated so that excess Cd was excreted in fecal balls, and the silkworm mortality rate was zero for all treatments. Therefore, the process of *Morus* growing, silkworm raising, and silk reeling and weaving can also help bring economic benefits to heavy metal- (HM-) contaminated areas, which will increase people’s acceptance of using *Morus* trees to remediate HM-contaminated soil.

Cd^2+^ is absorbed from the soil solution by root epidermal cells, and subsequently stored in part in the roots, with the remainder being translocated to the xylem vessels, and sequestered and detoxified in the vacuoles (Mendoza-Cózatl et al., 2011). Thus, uptake, translocation and sequestration of Cd are crucial plant processes for the phytoremediation of Cd-contaminated soil. However, Cd has no known biological function in plants, so there are no specific transporter systems for Cd in plants. Cd^2+^ is likely to be transported via transporters of essential divalent metal cations (such as Ca^2+^, Zn^2+^, and Fe^2+^) (Shahid et al., 2016). These transporters include the zinc-regulated transporters, iron-regulated transporter-like proteins (ZIP), the natural resistance and macrophage proteins (NRAMP), the heavy metal ATPases (HMA), and the metal tolerance or transporter proteins (MTP) family members, which have been functionally characterized mainly in *A. thaliana* and *Oryza sativa* (Luo et al., 2016).

Because of poor selectivity toward divalent metal cations (Fe^2+^, Mn^2+^, Cd^2+^, and Zn^2+^), NRAMP and ZIP family transporters are mainly responsible for Cd^2+^ absorption (Mendoza-Cózatl et al., 2011; Shahid et al., 2016). Some studies have reported that *N*. *caerulescens* NcZNT1, rice OsIRT1, OsIRT2, OsZIP6, *A. thaliana* AtIRT1, *Medicago truncatula* MtZIP6, OsNRAMP1, OsNRAMP5, and *Hordeum vulgare* NRAMP5 are all localized on the plasma membrane, while mediating Cd^2+^ uptake in different tissues (Vert et al., 2002; Nakanishi et al., 2006; Stephens et al., 2011; Takahashi et al., 2011; Ishimaru et al., 2012; Lin et al., 2016; Wu et al., 2016). Meanwhile, there are some members of the NRAMP family, localized to organellar membranes, which also play roles in the distribution of Cd^2+^ within the cell. AtNRAMP3 and AtNRAMP4 are localized to the tonoplast (Thomine et al., 2000; Lanquar et al., 2005), while AtNRAMP6 is targeted to a vesicular-shaped endomembrane compartment (Cailliatte et al., 2009).

Heavy metal ATPases belongs to the large P-type ATPase family, the members of which have been involved in transporting monovalent (Cu^+^/Ag^+^) and divalent (Ca^2+^/Zn^2+^/Cd^2+^/Co^2+^/Pb^2+^) HM cations. Many members of the latter group have been reported to transport Cd^2+^. AtHMA3 and OsHMA3 are located in the tonoplast, participating in Cd^2+^ sequestration to the vacuoles (Morel et al., 2009; Miyadate et al., 2011). Plasma membrane proteins OsHMA2, AtHMA2, and AtHMA4 can load Cd^2+^ into the xylem, playing a pivotal role in controlling Cd^2+^ translocation ability from the roots to the shoots of plants (Verret et al., 2004; Jarvis et al., 2009; Wong and Cobbett, 2009; Takahashi et al., 2012). AtHMA1 is localized to the inner envelope membrane of the chloroplast, and has been shown to be able to transport Cd^2+^ upon heterologous expression in *Saccharomyces cerevisiae* (Seigneurin-Berny et al., 2006; Moreno et al., 2008). MTP belongs to the Cation Diffusion Facilitator (CDF) family (Montanini et al., 2007). Most MTPs are localized to the tonoplast and act as antiporters of Zn^2+^, Ni^2+^, and Cd^2+^, which are involved in sequestration or efflux of these ions to reduce HM toxicity (Ovecka and Takac, 2014). In rice, overexpression of *OsMTP1* in yeast and tobacco conferred increased tolerance to Cd^2+^ (Yuan et al., 2012; Das et al., 2016). In *Arabidopsis*, AtMTP1 and AtMTP3 are involved in sequestration of excess Zn^2+^ (Arrivault et al., 2006; Kawachi et al., 2008).

Investigations into these four transporter families in woody plants are limited (Luo et al., 2016). In previous studies, we confirmed that two *Morus* phytochelatin synthase (PCS) genes conferred Zn/Cd tolerance and accumulation in transgenic *Arabidopsis* and tobacco (Fan et al., 2018). To further understand the mechanisms of Cd-phytoremediation, and to screen for key genes related to Cd uptake, translocation and sequestration in *Morus*, we identified nine ZIPs, four NRAMPs, 8 HMAs and 10 MTPs (31 genes in total) from *M. notabilis*. After CdCl_2_ treatment, expression analysis indicated that the corresponding genes from *M. alba* displayed various expression patterns, differing between tissues and with respect to the time of peak expression. Moreover, *MaIRT1, MaNRAMP1, MaZIP4*, and *MaHMA3* were heterologously expressed in *S. cerevisiae* to achieve functional characterization.

## Materials and Methods

### Identification of *ZIP, NRAMP, HMA*, and *MTP* Genes in *Morus*

Hidden Markov model (HMM) profiles (PF02535, PF01566, and PF01545) for domains corresponding to *ZIP, NRAMP*, and *MTP* gene families were downloaded from Pfam database^1^. The HMM profile of the *HMA* gene family was constructed using the hmmbuild feature from the HMMER package 3.1b2^2^ with aligned *A. thaliana HMA* and *O. sativa HMA* sequences from TAIR^3^ and Rice Information Resource^4^, respectively. The HMM search was performed using hmmsearch (using default *e*-value of 10) from the HMMER package on the mulberry proteome downloaded from the *M. notabilis* database^5^. Then, all *Morus ZIP, NRAMP, HMA*, and *MTP* genome sequences were directly downloaded from the *M. notabilis* database according to the Gene-IDs. The downloaded *ZIP, NRAMP, HMA*, and *MTP* candidate sequences were analyzed manually using the SMART^6^ and CDD^7^ databases for the presence of the domains. WoLF PSORT^8^ and ProtComp 9.0^9^ were used to predict protein subcellular localization. The TMHMM server^10^ was used to calculate the number of transmembrane helical (TM) domains.

### Phylogenetic Analysis, Exon–Intron Structure, Motif Analysis and Promoter *Cis-*Element Identification

Multiple sequence alignments of the full-length protein sequences were performed using the ClustalX version 2.1 program (Larkin et al., 2007), followed by manual adjustment using the BioEdit version 7.0.9 (Hall, 1999). MEGA version 5.05 (Tamura et al., 2011) was used to determine the substitution model and rate heterogeneity of the best fit with the ZIP, NRAMP, HMA and MTP protein data. Phylogenetic trees were constructed with a maximum likelihood method, using TREE-PUZZLE 5.3. rc16 (Steel, 2010). The number of puzzling steps was set at 10,000. Trees were displayed using online software iTOL^11^ (Letunic and Bork, 2016). The online software Gene Structure Display server 2.0^12^ (Hu et al., 2015) was used to generate the exon/intron organization of each *ZIP, NRAMP, HMA*, and *MTP* gene by comparing the cDNA to its corresponding genomic DNA sequence. The Multiple Expectation Maximization for Motif Elucidation (MEME) version 4.11.2^13^ was used to investigate conserved motifs for each *ZIP, NRAMP, HMA*, and *MTP* gene (Bailey et al., 2009). ZIP, NRAMP, HMA, and MTP family sequences from *A. thaliana, O. sativa* and other species were downloaded from TAIR, Rice Information Resource and the ARAMEMNON plant membrane protein database^14^, respectively. Promoter sequences, located 1500 bp upstream of the transcription start site, were searched using National Center for Biotechnology Information (NCBI)^15^, and were analyzed using the PlantCare database^16^ (Lescot et al., 2002).

### Plant Materials and Stress Treatments

Since *M. notabilis* seedlings only grow in Ya’an, Sichuan, China, at a height of ∼1400 m above sea level, and under ambient conditions such as a mean daytime temperature of ∼22°C, attempts to cultivate this species elsewhere were unsuccessful. *M. alba* cv. Guiyou No. 62 (Ma-GY62) has been used to study the expression profiles of *Morus* genes under different stresses (Yang et al., 2015; Cai et al., 2016). Therefore, we used seedlings of Ma-GY62 as the plant material for Cd^2+^ stress treatments. Ma-GY62 seedlings were planted in soil in a PQX-type plant incubator (Ningbo Southeast Instrument Corporation, Ningbo, China) with a 16 h/8 h light/dark cycle at 26°C/22°C (day/night). After approximately 2 months, the Ma-GY62 seedlings (∼15 cm high) were treated with 0 (control, CK), 30 or 100 μM CdCl_2_ solution, and the roots, stems, and leaves were sampled at 0, 1, 3, 10, or 24 h after treatment. The corresponding tissues of untreated seedlings were used as controls. All samples were instantly frozen in liquid nitrogen and stored at -80°C.

### Assessment of Cd Accumulation in Ma-GY62 Seedlings

To detect the Cd accumulation capacity of Ma-GY62 seedlings grown at the three soil concentrations of Cd (see section “Plant Materials and Stress Treatments”), the root, stem, and leaf tissue of seedlings were separated and immersed in 10 mM ethylenediamine tetra-acetic acid for 15 min and washed thoroughly three times with a neutral detergent solution and distilled water. Each sample was dried at 105°C for 30 min, followed by 75°C for 12 h. The dry tissues were ground and suspended in deionized water. The Cd concentrations were determined with atomic absorption spectrophotometry (VARIAN AA 240 Duo, Varian, Palo Alto, CA, United States). Each treatment consisted of three biological replicates.

### Cloning and Sequence Identification of Ma-GY62 ZIP, *NRAMP, HMA*, and *MTP* Genes

Total RNA from the different tissues was extracted using the RNAiso Plus Kit (TaKaRa, Dalian, China), following the manufacturer’s instructions. cDNA was synthesized from 1 μg total RNA in a 25 μl reaction volume using the PrimeScript RT reagent kit (TaKaRa). The gene-specific primers for the 31 genes were designed using Primer Premier 5.0 (Premier Biosoft International, Palo Alto, CA, United States) (**Supplementary Table S1**) to amplify the full-length coding sequences (CDS) of the genes from the total cDNA generated from Ma-GY62 leaves. The purified PCR products were TA cloned into the pMD^®^19-T simple vector (TaKaRa), and then were confirmed by sequencing.

### Expression Analysis of the *Morus ZIP, NRAMP, HMA*, and *MTP* Genes

To further investigate the expression of the 31 genes in different organs, the reads per kilobase of exon model per million mapped reads (RPKM) were used to compare differences in gene expression created by the MultiExperiment Viewer (Saeed et al., 2003). The root, branch bark, bud, flower, and leaf RPKM values of *M. notabilis ZIP, NRAMP, HMA*, and *MTP* genes were retrieved from RNA sequencing data^17^.

To confirm changes in expression of the 31 genes in response to Cd^2+^ stress, the gene expression profiles in root, stem, and leaves under different Cd^2+^ concentration treatments were analyzed by qPCR, with three replicates. The method of qPCR, using SYBR^®^ Premix Ex Taq^TM^ II (TaKaRa), was as described by Wei et al. (2014). The primer pairs used in the qPCR analysis of the 31 genes were designed using Primer Premier 5.0 (**Supplementary Table S1**). The *MaACTIN3* gene (HQ163775.1) was used as a control to normalize the relative expression of target genes. Relative expression was defined as 2^-[Cycle threshold (target gene)-*C*t (control gene)]^.

The data were normalized by log transformation. Cluster analysis was performed by using the pheatmap package version 1.0.8^18^ with Euclidean distance method created by the R package.

### Yeast Expression Analysis

The Cd-sensitive *ycf1* mutant strain Δ*Ycf1* (*BY4741*; *MAT*α*; his3*Δ*1; leu2*Δ*0; lys2*Δ*0; ura3*Δ*0; YDR135c::kanMX4*) and the Zn-sensitive *zrc* mutant strains Δ*zrc* (*BY4741; MATa; ura3*Δ*0; leu2*Δ*0; his3*Δ*1; met15*Δ*0; YMR243c::kanMX4*) were grown in YPD or SD-Ura synthetic dropout media. For overexpression of cDNAs of *MaIRT1, MaNRAMP1, MaZIP4*, or *MaHMA3*, the pYPGE15 yeast expression vector, carrying *URA3* as the selectable marker, was used (Cabello-Hurtado and Ramos, 2004). PCR primers, containing restriction enzyme cutting sites, were designed (**Supplementary Table S1**). After digestion with the corresponding restriction enzyme, they were cloned into pYPGE15.

Transformation of Δ*Ycf1* and Δ*zrc* strains with the target constructs were performed by electroporation (Becker and Guarente, 1991). For Zn/Cd tolerance testing, single colonies from SD-Ura agar plates were cultured in liquid SD-Ura medium until the optical density (OD_600_) reached 1.0. Serial dilutions (OD_600_ = 1, 0.1, 0.01, 0.001, 0.0001) were spotted onto SD-Ura agar containing various concentrations of either CdCl_2_ (10 or 20 μM) or ZnSO_4_ (5 or 10 mM). Plates were photographed after incubation at 30°C for 3 days. Aliquots (100 μl) of the foregoing yeast stains (OD_600_ = 1.0) were inoculated into 10 ml fresh liquid SD-Ura medium containing various concentrations of CdCl_2_ (10 or 20 μM) or ZnSO_4_ (5 or 10 mM). The OD_600_ values were measured every 2 h over a 30-h incubation period.

### Subcellular Localization in Yeast and Tobacco Leaves

pYPGE15-EGFP were used to observe the localization of MaIRT1, MaNRAMP1, MaZIP4 or MaHMA3 in yeast. The overexpression vector pZYGC [transformed from PLGNL by Lv Z, which contains the cauliflower mosaic virus 35S promoter, enhanced green fluorescent protein (EGFP) and terminator] was used to observe the localization of those four transporter in tobacco (*Nicotiana benthamiana*). PCR primers, without the stop codon but containing restriction enzyme cutting sites, were designed (**Supplementary Table S1**).

These yeast constructs were introduced into the Δ*Ycf1* strain. Then, those yeast strains were cultured in liquid SD-Ura medium until the OD_600_ reached 1.0. The new constructs of pZYGC-*MaIRT1*, -*MaNRAMP1*, -*MaZIP4*, -*MaHMA3* were transformed into *Agrobacterium tumefaciens* strain GV3101 via a freeze-thaw method (Chen et al., 1994). Then, those *A. tumefaciens* strains were injected into the tobacco leaf lamina for transient expression of EGFP (Sparkes et al., 2006; Luo et al., 2018). Fluorescent cells were imaged by FV1200 laser scanning confocal microscopy (Olympus, Tokyo, Japan).

### Statistical Analysis

Analysis of the data was carried out using parametric ANOVA, with multiple pairwise comparisons being carried out by Tukey’s test (*P* < 0.05). The data were analyzed and performed by Excel (Microsoft, Redmond, WA, United States), SPSS Statistics for Windows, version 17.0 (SPSS Inc., Chicago, IL, United States) and GraphPad Prism version 6.0 (GraphPad Software, San Diego, CA, United States).

## Results

### Identification and Bioinformatics Analysis of *ZIP, NRAMP, HMA*, and *MTP* Genes From *M. notabilis*

We identified 9 *MnZIP*s, four *MnNRAMP*s, eight *MnHMA*s and 10 *MnMTP*s (a total of 31 genes) from the *M. notabilis* genome (**Table 1** and **Supplementary Table S2**), and the number of members of these four gene families in the *Arabidopsis* and rice genomes, according to previous research, are also shown in **Supplementary Table S2**.

**Table 1:** *ZIP, NRAMP, HMA* and *MTP* genes in the *Morus notabilis* genome.

Gene name	GenBank accession numbers	Genomic position^a^	CDS length (bp)	Amino acid number	TM domains predicted^b^	Protein subcellular localization predicted^c^
*MnIRT1*	MG773132	scaffold73:955306-957300:+	1056	351	8	P
*MnIRT2*	MG773131	scaffold923:142877-145960:+	1077	358	7	P
*MnZIP1*	MG773133	scaffold96:597491-600594:-	1056	351	7	P
*MnZIP2*	MG773139	Scaffold46:662828-664781:-	996	331	9	P
*MnZIP3*	MG773137	scaffold46:672440-675545:-	1254	417	11	P
*MnZIP4*	MG773135	scaffold804:172706-174858:+	1221	406	8	P
*MnZIP5*	MG773134	scaffold611:86590-89167:-	1077	358	7	P
*MnZIP6*	MG773136	scaffold609:23407-26602:-	1095	364	6	P
*MnZIP7*	MG773138	scaffold1436:239650-241454:-	1041	346	8	P
*MnNRAMP1*	MG773143	scaffold410:250368-254753:-	1530	509	10	P
*MnNRAMP2*	MG773141	scaffold286:78033-81145:+	1596	531	12	V
*MnNRAMP3*	MG773140	scaffold1633:86406-89064:+	1569	522	12	V
*MnNRAMP4*	MG773142	scaffold1379:244650-249730:-	1536	511	10	P
*MnHMA1*	MG773147	scaffold939:110033-116937:-	2496	831	5	Ch
*MnHMA2*	MG773144	scaffold640:168074-174152:+	2847	948	7	P
*MnHMA3*	MG773150	scaffold297:742794-749609:-	2970	989	8	P, G
*MnHMA4*	MG773145	scaffold1005:74488-79507:+	2901	966	8	P, G
*MnHMA5*	MG773149	scaffold297:730431-736662:-	2955	984	6	P, G
*MnHMA6*	MG773151	scaffold1219:351792-370928:+	2853	950	3	Ch
*MnHMA7*	MG773146	scaffold4905:16794-23010:-	3000	999	7	P, G
*MnHMA8*	MG773148	scaffold348:210110-217043:+	2691	896	3	Ch
*MnMTP1*	MG773160	scaffold1303:555709-556884:-	1176	391	6	V
*MnMTP2*	MG773153	scaffold650:70488-75395:-	1143	380	0	M
*MnMTP3*	MG773153	scaffold789:836249-837526:-	1293	430	6	V
*MnMTP4*	MG773153	scaffold46:1049130-1050209:+	1086	361	6	V
*MnMTP5*	MG773153	scaffold139:891910-895885:+	1185	394	4	V
*MnMTP6*	MG773153	scaffold435:167772-172266:-	1227	408	5	G, V
*MnMTP7*	MG773153	scaffold2452:8263-14877:-	1353	450	4	G, V
*MnMTP8*	MG773153	scaffold989:550983-553310:-	1230	409	5	G, V
*MnMTP9*	MG773153	scaffold179:147396-151647:-	1095	364	5	G, V
*MnMTP10*	MG773153	scaffold179:132860-139500:-	1272	423	4	G, V

*^*a*^Data from MorusDB. ^*b*^Number of transmembrane helices predicted with TMHMM Server. ^*c*^WoLF PSORT and ProtComp 9.0 predictions: P (plasma membrane), V (vacuolar membrane), Ch (chloroplast), M (mitochondrion) and G (Golgi apparatus).*

The identified *MnZIP*s encoded proteins that varied in length from 331 to 416 amino acids (AAs), and contained 6–11 transmembrane (TM) domains predicted with the TMHMM Server. MnZIPs also have a variable region between the TM-3 and TM-4 variable regions, containing a potential metal-binding domain, which is rich in conserved histidine residues (**Supplementary Figure S1**). The identified *MnNRAMP*s encoded proteins that varied in length from 509 to 531 AAs, and contained 10–12 TM domains. The identified *MnHMA*s encoded proteins that varied in length from 831 to 999 AAs, and contained 3–8 TM domains. The identified *MnMTP*s encoded proteins that varied in length from 361 to 450 AAs, and contained 4–6 TM domains, except for *MnMTP2*, which was predicted to lack a TM domain.

We used WoLF PSORT and ProtComp 9.0 to predict the location of each protein in the cell. These results showed that all MnZIPs, MnNRAMP1, 4 and MnHMA2 were localized to the plasma membrane; MnNRAMP2-3, MnMTP1, 3–5 were localized to the vacuolar membrane; MnHMA3-5 and 7 were localized to both the plasma membrane and the Golgi apparatus; MnMTP6-10 were localized to both the Golgi apparatus and the vacuolar membrane; while MnHMA1, 6, and 8 were localized to the chloroplast, with MnMTP2 being localized to the mitochondrion.

### Phylogenetic Analyses, Classification and Functional Relatedness of the *ZIP, NRAMP, HMA*, and *MTP* Genes

**FIGURE 1:**
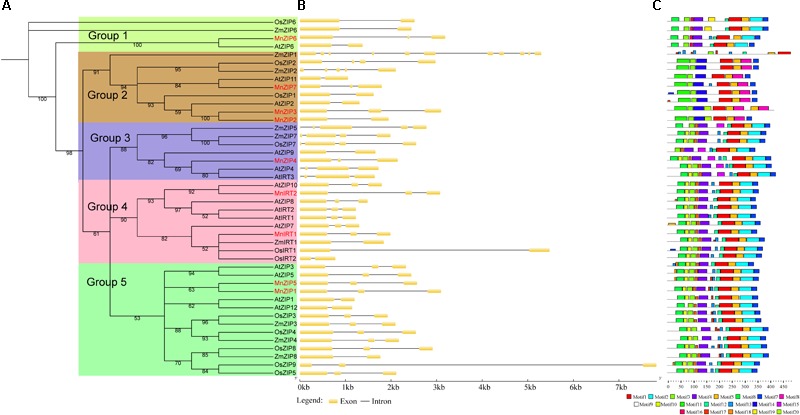
Phylogenetic relationships, gene structure and motif composition of *ZIP* genes in *Arabidopsis* (At), rice (Os), *Morus* (Mn) and other species. **(A)** Multiple sequence alignments of the full-length protein sequences of *ZIP* genes were performed using ClustalX version 2.1. Phylogenetic trees were constructed with the maximum likelihood method using TREE-PUZZLE 5.3. rc16. The number of puzzling steps was set at 10,000 times. Every colored box represents a subfamily. **(B)** Exon/intron structures. Yellow boxes and black lines represent exons and introns, respectively. **(C)** Schematic representation of the conserved motifs elucidated by MEME 4.11.2. A number in the colored box represents each motif, and the black lines represent the non-conserved sequences.

To examine the phylogenetic relationships among the *ZIP, NRAMP, HMA*, and *MTP* genes from *A. thaliana, O. sativa, M. notabilis* and other species, we performed phylogenetic analyses of the ZIP, NRAMP, HMA, and MTP protein sequences based on a maximum likelihood method, using TREE-PUZZLE 5.2 (**Figure 1** and **Supplementary Data Sheet S1**). ZIPs were categorized into five groups (Groups 1–5), according to their phylogenetic relationships. NRAMPs were divided into two groups. HMAs were divided into two groups: Group 1 (Zn/Cd/Co/Pb) and Group 2 (Cu/Ag). MTPs were categorized into seven groups: Groups 1, 5, and 12 belonged to the Zn-CDFs, Groups 8 and 9 belonged to the Mn-CDFs, and Group 1 and Groups 6–9 belonged to the Fe/Zn-CDFs. In general, each group contained members of the transporter families from *Morus, Arabidopsis*, and rice. It is possible that these groups had evolved before the monocot-dicot divergence. There were four paralogous pairs: *MnZIP1*/*MnZIP5, MnZIP2*/*MnZIP3, MnHMA3*/*MnHMA5*, and *MnMTP9*/*MnMTP10*. The latter three pairs were localized to the same scaffold.

The CDS of the *MnZIP* genes were disrupted by 1–2 introns. There were three introns in *MnNRAMP* Group 2, compared to 11 in Group 1. The number of introns in the *MnHMA* genes ranged from 5 to 16, while the number of introns in the *MnMTP* genes ranged from 5 to 12. Comparison of the exon/intron organization of the individual members of the four gene families showed that most of the closely related members shared similar structures, with regard both to the number of introns and the exon length. These results were highly consistent with the characteristics described in the above phylogenetic analysis.

The results of conserved domain analysis (data not shown) performed with SMART, Pfam and CDD showed that all ZIP proteins possessed one conserved ZIP domain, while all NRAMP proteins possessed one conserved NRAMP domain essential for their transporter activity. All HMA proteins in the foregoing species possessed one E1-E2_ATPase hydrolase domain. Members of Group 2 of the HMA proteins possessed HM-associated domains, and the number of these domains present in different clades ranged from 1 to 3. All MTP proteins in the above species possessed one cation efflux domain. The Group 12, Group 6, Group 8, and Group 9 MTPs in the above species possessed one ZT dimer domain.

In order to identify smaller individual motifs and more divergent patterns, we used the MEME program to study the diversity of *ZIP, NRAMP, HMA*, and *MTP* genes in *Morus, Arabidopsis*, rice, and other species. Details of the 20 motifs of the four transporter families are presented in **Supplementary Data Sheet S2**. In general, the number of motifs in the four transporter family proteins in *Morus* and the number of AAs spacing between adjoining protein motifs differed, a finding which was similar to that observed in the *Arabidopsis* and rice proteins.

There were common motifs among the most closely related members in the phylogenetic tree. Among the 44 *ZIP* sequences, more than 40 *ZIP* genes contained motifs 1, 3, 5, and 7. Group 2 *ZIP* sequences were different from the other Groups, containing motifs 8, 11, and 14, but lacking 2, 4, and 6. Among the 21 *NRAMP* sequences, more than 20 *NRAMP* genes contained motifs 1–9, 11, and 12. Compared with Group 1, Group 2 *NRAMP* genes contained motifs 10, 14, 16, and 17, but lacked motifs 13 and 15. Among the 37 *HMA* sequences, more than 30 *HMA* genes contained motifs 1–5, 8–11, 16, 18, and 19. Compared with Group 1, Group 2 *NRAMP*s contained motifs 6–7, 13–14, and 20, which may be related to their role in Cu/Ag transport. Among the 31 *MTP* sequences, the motifs of members of Groups 8 and 9 were similar, with 26 *MTP* genes containing motif 5. Some AA sites of these motifs may play important roles in the transport of cadmium or other ions. Among the selected yeast mutations expressing *AtNRAMP4*, three residues (L67V, E401K, and F413I) decreased Cd^2+^ sensitivity (Pottier et al., 2015).

### Promoter *Cis*-Element Analysis

As primary signaling molecules, phytohormones such as jasmonic acid (JA) and methyl jasmonate (MeJA), salicylic acid (SA), auxins, gibberellins (GA), ethylene (ET), and abscisic acid (ABA) (Saeed et al., 2003) enable plants to withstand abiotic stresses, including HMs (Luo et al., 2016). We identified putative *cis*-acting regulatory DNA elements based on the promoter sequences acquired (1500 bp upstream of the transcription start site). Analysis of the promoter sequences of members of the four gene families identified several *cis*-elements that were related to responsiveness to phytohormones and environmental stress signals (**Supplementary Table S3**). Among the 31 genes, 28 genes (90.32%) contained ABA-responsive *cis*-elements (ABRE/ARE), 22 genes (70.97%) contained SA-responsive *cis*-elements (TCA-element), 14 genes (45.16%) contained MeJA-responsive *cis*-elements (CGTCA-motif/TGACG-motif) and eight genes (25.81%) contained auxin-responsive *cis*-elements (TGA-element), while 11 genes (35.48%) contained GA-responsive *cis*-elements (GARE-motif). In addition, there were several genes where the promoter contained MYB-binding sites involved in drought-inducibility (MBS), heat stress-responsive and wounding- and pathogen-responsive motifs (W box/WUN-motif). All genes, except *MnZIP2*, contained more than three classes of *cis*-elements, which shows the diversity of the expression regulatory sequences associated with these genes.

### Differential Expression Profiles of the *ZIP, NRAMP, HMA*, and *MTP* Genes

**FIGURE 2:**
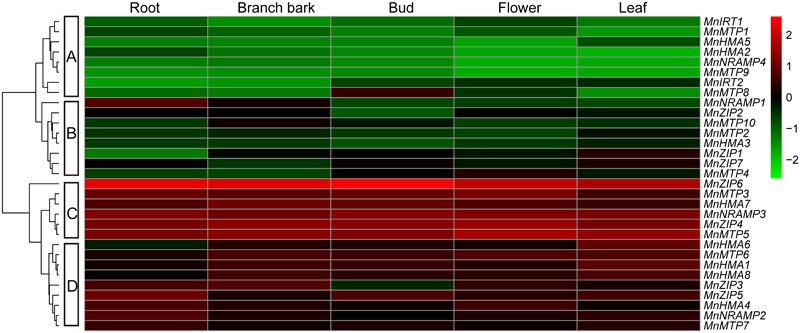
The transcript levels of four *Morus* transporter gene families in five tissues. The transcript levels were depicted by color scale representing log10 values. The values were centered and scaled in the column direction. Red denotes high expression and green denotes low expression.

The RPKM was used to normalize the expression levels of the *MnZIP*s, *MnNRAMP*s, *MnHMA*s, and *MnMTP*s in five tissues (root, branch bark, bud, leaf, and flower), using *M*. *notabilis* RNA sequencing data^19^. Most genes showed different transcript levels among the five tissues, while the expression of *MnNRAMP4* and *MaMTP9* in buds and flowers, and the expression of *MnHMA2* in leaf tissue were not detected (**Figure 2**). The transcript levels of the 31 genes can be classified into four groups (A–D; **Figure 2**), with the transcript abundance decreasing generally in the order C > D > B > A. Because some transporters have the same functions of Cd^2+^ transport, perhaps the genes which showed low expression in the five tissues do not play important roles in Cd^2+^ transport.

**FIGURE 3:**
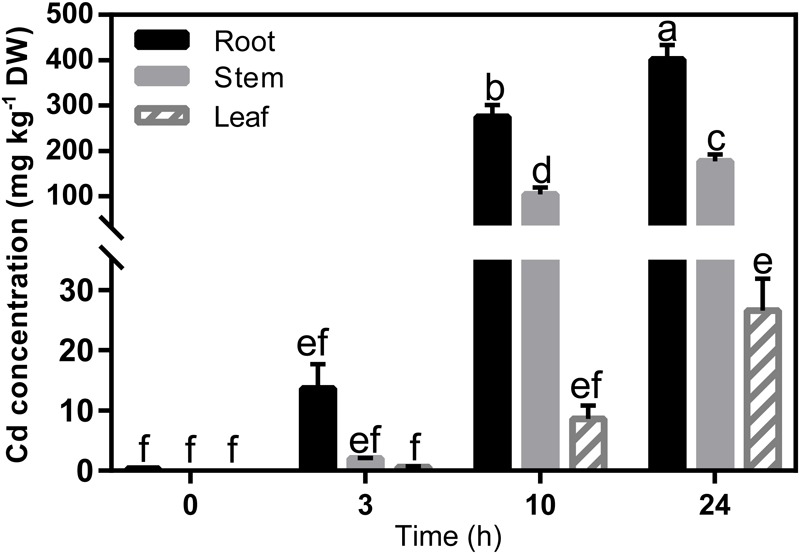
Cd concentration in root, stem and leaf tissues of Ma-GY62 seedlings within 24 h of exposure to soil treated with 100 μM CdCl_2_. Values are the means ± standard error (*n* = 3) for the different parts of the plant. Any two samples with a common letter are not significantly different, according to Tukey’s test (*P* > 0.05).

**FIGURE 4:**
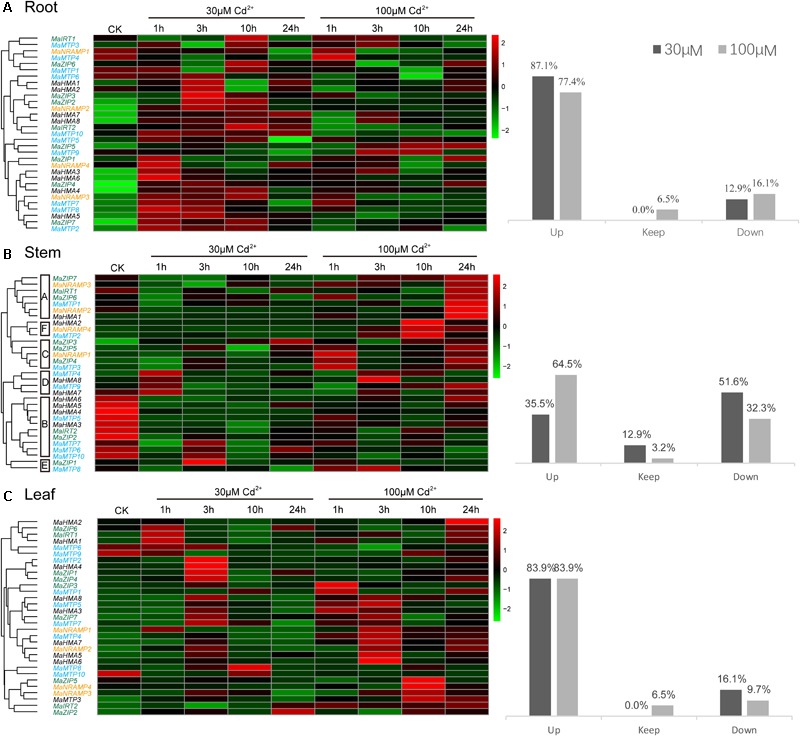
The transcript levels of four transporter gene families in **(A)** root, **(B)** stem, and **(C)** leaf tissues of Ma-GY62 under Cd^2+^ stress. The transcript levels were depicted by color scale representing log10 values. The values were centered and scaled in the row direction. Red denotes high expression and green denotes low expression. The percentage of up-regulation, no significance difference, or down-regulation of the 31 genes in root, stem, and leaf, in the presence of 30 or 100 μM Cd^2+^, is to the right hand side of the pheatmap.

The Cd concentration in the seedlings increased significantly in all tissues within 24 h of exposure to Cd (**Figure 3**), which means that the translocation of Cd was very rapid. The expression profiles of the 31 genes in Ma-GY62 under Cd^2+^ stress were then compared (**Figure 4**). The roots, stems, and leaves were sampled at 0, 1, 3, 10, and 24 h after Cd^2+^ treatment. We cloned the CDS of the Ma-GY62 *ZIP, NRAMP, HMA*, and *MTP* genes, and the AA sequence identities between the Cd^2+^ transporters of *M. notabilis* and Ma-GY62 were high (≥87%) (**Supplementary Table S4**).

In roots, 87.1% of the 31 genes showed increased expression within 24 h of exposure to the low Cd^2+^ concentration (30 μM) treatment, but the time of peak up-regulation was different among the various genes. The expression of *MaZIP1, 7, MaNRAMP4, MaHMA3, 6, MaMTP2*, 5, 7-8 and 10 (37% of the total of 31 genes) reached the maximum at 1 h, while the expression of *MaZIP2*-*3, 5*-*6, MaNRAMP2, MaHMA1*-*2, 5*-*6* (33.3%) was maximal at 3 h. The expression of genes *MaIRT1*-*2, MaZIP6, MaNRAMP3, MaHMA4, MaHMA8, MaMTP3* (25.9%) reached the maximal value at 10 h, while the expression of *MaHMA7* (3.7%) peaked at 24 h. The expression of genes *MaNRAMP1, MaMTP1, 4* and *9* (12.9%) decreased within 24 h of exposure to Cd^2+^.

When the studies were repeated at the high Cd^2+^ concentration (100 μM), 77.4% of the 31 genes showed increased expression within 24 h, but, again, the times of maximal up-regulation were different. The expression of *MaZIP1, 3, MaNRAMP2*-*3, MaHMA4, MaMTP4*-*5* and *7* (25.8% of the total of 31 genes) reached their maximum at 1 h, while that of *MaIRT1, MaNRAMP4, MaHMA3, MaHMA7*-*8, MaMTP3* and *9* (22.6%) peaked at 3 h, with the expression of *MaZIP5* and *MaMTP2* (6.5%) reaching the maximum at 10 h, and that of *MaZIP4, 6-7, MaHMA1*-*2* and *5* (19.4%) peaking at 24 h. The expression of genes *MaIRT2, MaNRAMP1, MaMTP1, 6* and *10* (16.1%) showed decreased expression over the 24-h exposure period, while *MaZIP2* and *MaHMA6* (6.5%) showed no significant difference in expression in response to high Cd^2+^ concentration (100 μM). The maximum expression of those genes which showed increased expression under 30 μM Cd^2+^ stress was higher than that under 100 μM Cd^2+^ stress except for genes *MaZIP5* and *MaHMA8*. *MaNRAMP1* and *MaMTP1* showed decreased expression under both 30 and 100 μM Cd^2+^ stress, while *MaIRT2, MaMTP6*, and *MaMTP10* showed increased expression after 30 μM Cd^2+^ treatment and decreased expression after 100 μM Cd^2+^ treatment. In contrast, *MaMTP4* and *MaMTP9* showed decreased expression after 30 μM Cd^2+^ treatment but increased expression after 100 μM Cd^2+^ treatment.

In stems (**Figure 4B**), 35.5% of the 31 genes studied showed increased expression within 24 h of Cd exposure to the low Cd^2+^ concentration (30 μM) treatment, but the time of maximal expression differed between genes. The expression of *MaHMA7*-*8, MaMTP4* and *9* (12.9%) was maximal at 1 h, while that of *MaZIP1, 4-5, MaMTP2* and *7* (16.1%) peaked at 3 h. Genes *MaZIP3* and *MaNRAMP1* (6.5%) reached maximal expression at 24 h, while genes *MaIRT1-2, MaZIP2, 6-7, MaNRAMP2-3, MaHMA3-6, MaMTP1, 5-6, 8* and *10* (51.6%) showed decreased expression during Cd^2+^ exposure, and genes *MaNRAMP4, MaHMA1*-*2, MaMTP3* (12.9%) showed no significant difference in expression in response to Cd^2+^ exposure. At high Cd^2+^ concentration (100 μM) treatment, 64.5% of the 31 genes in stem tissue showed increased expression within 24 h, but the time of maximal up-regulation differed. The expression of genes *MaZIP1, MaNRAMP1* and *MaMTP3* (9.7%) was maximal at 1 h, while that of *MaHMA8, MaMTP4* and 8 (9.7%) peaked at 3 h. The expression of *MnZIP3, MaNRAMP4, MaHMA2 and MaMTP2* (12.9%) reached the maximum level at 10 h, while that of *MaIRT1, MaZIP4-7, MaNRAMP2, MaHMA1, 7, MaMTP1* and *9* (32.3%) peaked at 24 h. Genes *MaIRT2, MaZIP2, MaHMA3-6, MaMTP5-7* and *10* (32.3%) showed decreased expression over the 24 h period of exposure, while that of gene *MaNRAMP3* (3.2%) showed no significant difference. The genes of Group B showed decreased expression under both 30 and 100 μM Cd^2+^ treatments except for *MaMTP7*, which showed increased expression after 30 μM Cd^2+^ treatment and decreased expression after 100 μM Cd^2+^ treatment. The genes of Groups C, D, and E, which showed increased expression after 30 μM Cd^2+^ treatment, also showed increased expression after 100 μM Cd^2+^ treatment. Genes *MaNRAMP4* and *MaHMA1-2* showed no significant difference in expression after 30 μM Cd^2+^ treatment but expression increased after 100 μM Cd^2+^ treatment. Interestingly, genes *MaIRT1, MaZIP6-7, NRAMP2, MaMTP1* and *8* showed decreased expression after 30 μM Cd^2+^ treatment but increased expression after 100 μM Cd^2+^ treatment.

In leaves (**Figure 4C**), 83.9% of the 31 genes showed increased expression within 24 h of exposure to the low Cd^2+^ concentration (30 μM) treatment, but the time of maximal up-regulation differed between genes. The expression of genes *MaIRT1, MaZIP6, MaNRAMP1, MaHMA1* and *2* (16.1%) peaked at 1 h, compared to the expression of *MaZIP1, 3-4, 7, MaNRAMP2-3, MaHMA3-8, MaMTP2-5* and *7* (51.6%), which reached the maximal level at 3 h, while expression of *MaMTP1* and *8* (6.5%) peaked at 10 h and that of *MaIRT2* and *MaZIP2* (6.5%) was maximal at 24 h. Meanwhile, genes *MaZIP5, MaNRAMP4, MaMTP6, 9* and *10* (16.1%) showed decreased expression over the 24 h period. After treatment with high Cd^2+^ concentration (100 μM), 83.9% of the 31 genes showed increased expression within 24 h (the same proportion as with the low Cd^2+^ concentration (30 μM) treatment), but the time of maximal up-regulation was different. The expression of *MaZIP3, MaHMA1, 3, MaMTP1* (12.9%) was maximal at 1 h while the expression of genes *MaZIP7, MaNRAMP1*-*2, MaHMA5*-*8, MaMTP4*-*5, 7* (29%) peaked at 3 h, and that of genes *MaZIP2-3, 5, MaNRAMP3-4* and *MaMTP3* (19.4%) was maximal at 10 h. The expression of genes *MaIRT1*-*2, MaZIP1, 4-5, MaHMA2, MaMTP2* (22.6%) peaked at 24 h. Meanwhile, genes *MaMTP6, 9* and *10* (9.7%) showed decreased expression within the 24 h treatment period, and expression of *MaHMA4 and MaMTP8* (6.5%) showed no significant difference over this period. *MaMTP6, 9-10* showed decreased expression under both 30 and 100 μM Cd^2+^ treatments. In contrast, 77.4% of genes showed increased expression under both 30 and 100 μM Cd^2+^ treatments. Interestingly, genes *MaZIP5* and *MaNRAMP4* showed decreased expression after 30 μM Cd^2+^ treatment but increased expression after 100 μM Cd^2+^ treatment. On the other hand, *MaHMA4* and *MaMTP8*, which showed decreased expression after 30 μM Cd^2+^ treatment, showed no significant difference after 100 μM Cd^2+^ treatment.

### Heterologous Expression of Genes *MaNRAMP1, MaHMA3, MaZIP4*, and *MaIRT1* in *S. cerevisiae*

Roots and leaves, which accumulated the highest amounts of the Cd taken up, can be harvested, baled, dried, and incinerated to achieve phytoremediation. Therefore, we selected several genes, which were highly expressed in root or leaf tissues, and which showed significant increases or decreases in response to Cd treatment. Under both Cd concentrations, *MaNRAMP1* showed decreased expression in roots. Under 30 and 100 μM Cd^2+^ stress, *MaHMA3* expression increased 22- and 14-fold, respectively, in the root, while that of *MaZIP4* increased 10.5- and 9.7-fold, respectively, in the root. Moreover, *MaIRT1* increased 20- and 43-fold under 30 and 100 μM Cd^2+^ stress, respectively, in leaves. These four transporter genes were predicted to be located in the plasma membrane.

**FIGURE 5:**
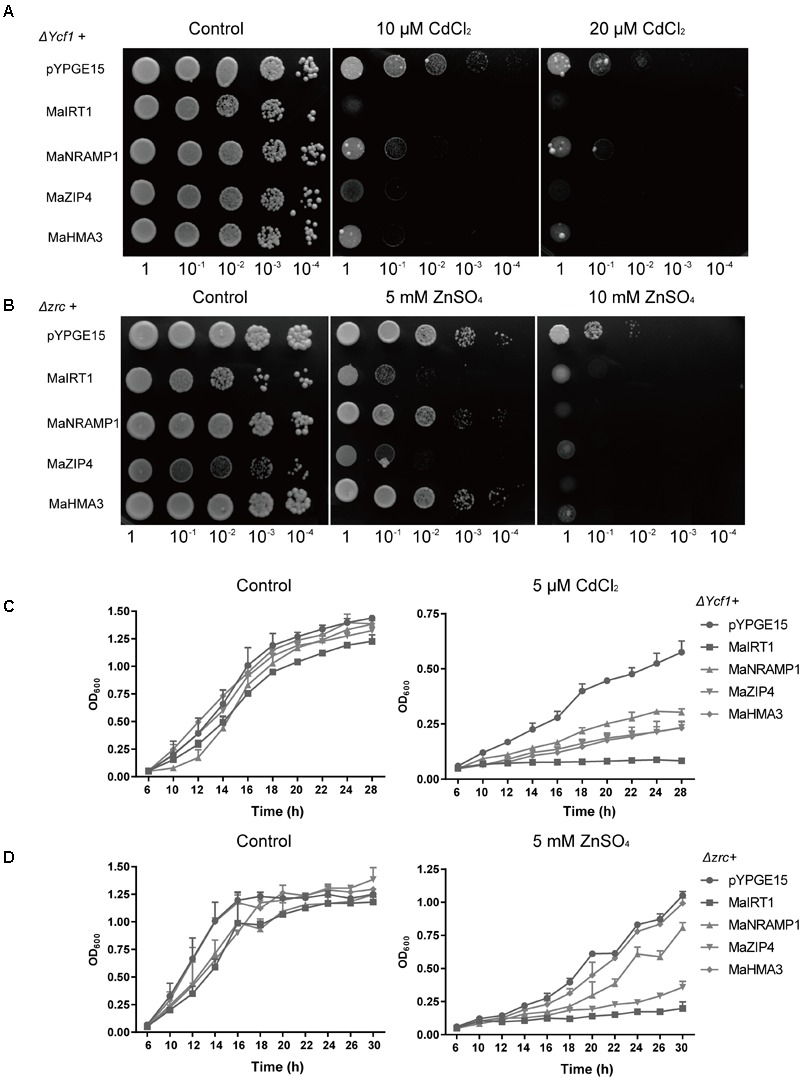
Functional analysis of Ma-GY62 *IRT1, NRAMP1, ZIP4* and *HMA3* expression in yeast strains. **(A)** Heterologous expression of genes *MaIRT1, MaNRAMP1, MaZIP4*, or *MaHMA3* increased the Cd sensitivity of the Δ*Ycf1* strain. Growth of yeast strains transformed with the empty vector (pYPGE15) or *MaIRT1, MaNRAMP1, MaZIP4* or *MaHMA3* on synthetic defined medium with 0, 10, or 20 μM CdCl_2_ was assessed. **(B)** Expression of *MaIRT1* or *MaZIP4* increased the Zn sensitivity of the Δ*zrc* strain. Growth of yeast strains transformed with the empty vector (pYPGE15) or with the genes *MaIRT1, MaNRAMP1, MaZIP4*, or *MaHMA3* on synthetic defined medium with 0, 5, or 10 mM ZnSO_4_ was assessed. Yeast cells transformed with the empty vector or with genes *MaIRT1, MaNRAMP1, MaZIP4*, or *MaHMA3* were grown in liquid SD medium containing **(C)** 5 μM CdCl_2_ or **(D)** 5 mM ZnSO_4_ for 30 h.

To investigate whether MaIRT1, MaNRANP1, MaZIP4, and MaHMA3 can transport Cd^2+^ and/or Zn^2+^, the cDNA of each of these four genes was heterologously expressed in yeast (Δ*Ycf1* and Δ*zrc* strains) for functional assays. All strains of yeast expressing *MaIRT1, MaNRANP1, MaZIP4*, or *MaHMA3* displayed considerably increased sensitivity to Cd (**Figures 5A,C**), whereas the expression of *MaNRAMP1* and *MaHMA3* did not alter the growth of yeast on medium containing 5 mM ZnSO_4_ (**Figures 5B,D**). These results suggest that MaIRT1 and MaZIP4 can transport both Zn^2+^ and Cd^2+^ into the yeast cell, while MaNRAMP1- or MaHMA3-mediated transport was more specific for Cd^2+^.

### MaIRT1, MaNRAMP1, MaZIP4, and MaHMA3 Are Localized to the Plasma Membrane of Yeast and Tobacco

**FIGURE 6:**
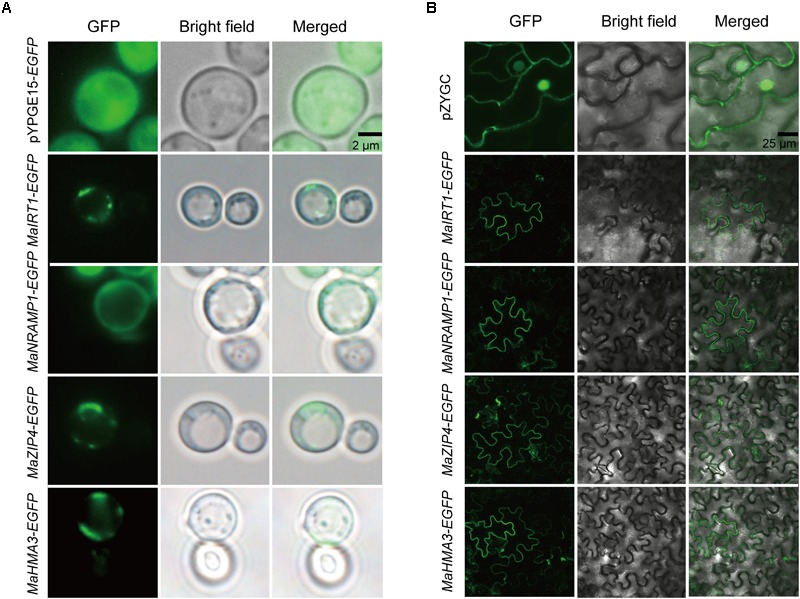
Subcellular localization of MaIRT1, MaNRAMP1, MaZIP4, and MaHMA3 in yeast and tobacco leaves. **(A)** Analyses of subcellular localization of the four transporter proteins in yeast strain Δ*Ycf1*. pYPGE15-*EGFP* means the empty vector. Scale bar = 2 μm. **(B)** Analyses of subcellular localization of those four transporter proteins in tobacco (*Nicotiana benthamiana*) leaf lamina. pZYGC means the empty vector. Scale bar = 25 μm.

To further determine whether the subcellular localization of MaIRT1, MaNRAMP1, MAZIP4, and MaHMA3 is consistent with the predictions, and whether the result of subcellular localization in yeast is consistent with that in plants, the subcellular localization of these four transporter proteins was investigated using an EGFP fusion protein in yeast and tobacco (**Figure 6**). Compared with the controls, MaIRT1, MaNRAMP1, MaZIP4, and MaHMA3 are all localized to the plasma membrane in both the yeast and tobacco cells.

## Discussion

### Multigene Family Members May Be Related to Functional Redundancy

**FIGURE 7:**
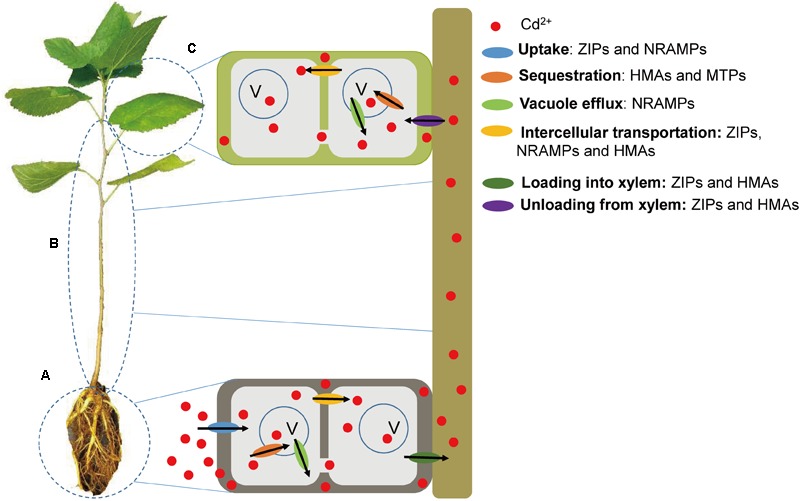
The mechanisms of Cd^2+^ uptake, translocation and sequestration in mulberry may be mediated by ZIP, NRAMP, HMA, and MTP transporters. **(A)** At the root level, ZIP and NRAMP transporters are involved in Cd^2+^ uptake. HMA and MTP transporters are involved in Cd^2+^ sequestration into vacuole. Vacuole efflux is mediated by NRAMP transporters. Intercellular transportation is mediated by ZIP, NRAMP, and HMA transporters. **(B)** At the stem level, loading of Cd^2+^ into xylem and unloading of Cd^2+^ from xylem are mediated by ZIP and HMA transporters. **(C)** At the leaf level, HMA and MTP transporters are involved in Cd^2+^ sequestration into vacuole. Vacuole efflux is mediated by NRAMP transporters. Intercellular transportation is mediated by ZIP, NRAMP, and HMA transporters.

Cadmium is initially absorbed from the soil through the roots, and then proportions of the Cd^2+^ are either entrapped in root vacuoles or enter the shoot via the xylem. Cd^2+^ in the xylem of the stem can then be either transported to the leaves or redistributed through the phloem (Mendoza-Cózatl et al., 2011). Enhanced active metal transport, rather than metal complexation, is the key mechanism of hyperaccumulation, which can result in accumulation of high concentrations of HMs in aerial parts of plants (Leitenmaier and Kupper, 2013). In the present study, we identified 31 *Morus* genes, including *ZIP, NRAMP, HMA*, and *MTP* genes, which may be involved in Cd^2+^ uptake, translocation and sequestration in *Morus* (**Figure 7**). These multigene families are possibly adapted to the transport of various ions. Functional redundancies may also exist among these metal transporters. The expected side effects were minor in the transformants or mutants of *OsHMA3*, probably due to compensation by other transporters (Miyadate et al., 2011; Sasaki et al., 2014). *MnZIP2*/*MnZIP3, MnHMA3*/*MnHMA5*, and *MnMTP9*/*MnMTP10* were localized to the same scaffolds, and are related as a result of tandem duplications. Although OsNRAMP1 and OsNRAMP5 both play roles in root Cd uptake, the contribution of OsNRAMP1 is probably insignificant compared to that of OsNramp5. *OsNRAMP1* expression in roots is much lower than *OsNRAMP5*, while increased *OsNRAMP1* expression did not increase root Cd uptake in the *OsNRAMP5* mutant (Uraguchi and Fujiwara, 2013). Moreover, the transcript abundance of the 31 genes in the five tissues of *Morus* differed greatly and probably decreased in the order Group C > D > B > A. The higher expression of a gene may suggest a greater role for this gene in Cd^2+^ transport processes.

### The Molecular Mechanism of Transporter Response to Cd^2+^ Stress in Roots

Cd^2+^ enters root cells via ZIP (OsIRT1, OsIRT2) and NRAMP (TcNRAMP3) transporters (Nakanishi et al., 2006; Wei et al., 2009). Cd^2+^ can also enter plant root cells via yellow-stripe 1-like proteins as a Cd-chelate complex (Curie et al., 2009). *MaNRAMP1* and *MaMTP1* showed reduced expression in roots under both 30 and 100 μM Cd^2+^ stress. *Morus* NRAMP1 was localized to the plasma membrane, and the expression of *MnNRAMP1* was higher in roots than in other tissues. *Morus* MTP1, MTP4 and MTP9 belonged to Groups 1 and 9 in the MTP phylogenetic tree (**Supplementary Data Sheet S1**), in which the same group members were reported to transport Cd^2+^ (Yuan et al., 2012; Das et al., 2016). Cd^2+^ is harmful to the growth of plants, so, for the plant itself, the expression of transporters involving Cd uptake in the root would logically be decreased, and that of transporters involving Cd transport and sequestration in root cells would be increased to reduce the toxicity of Cd. Moreover, because of the similarities in chemical properties and interrelated metabolic pathways, there is a close correlation between Cd and Zn/Cu/Co/Mg (Feng et al., 2017). Due to the Cd-mediated competitive inhibition of the uptake and transport of Zn^2+^ or other ions, some genes would exhibit increased expression to regulate the balance of the essential elements they need. In this study, the genes which exhibited Cd^2+^-induced increase in expression were members of all four transporter families. Of those genes, the expression of *MaHMA3* increased 22- and 14-fold under 30 and 100 μM Cd^2+^ stress, respectively, suggesting an important role in Cd transport in root cells, while the response of *MaZIP4* expression (10.5- and 9.7-fold increases under low and high Cd concentrations, respectively) indicates that it may play a similar role.

Cd^2+^ uptake in poplar roots was associated with activities of plasma membrane proton ATPases, and with the increased expression levels of several putative metal transporter genes, such as *NRAMP1.1, NRAMP1.3, ZIP2*, and *ZIP6.2* (He et al., 2015). Rome et al. (2016) also reported that *P. alba NRAMP1, IRT1a* and *IRT1b* genes increased expression in roots after 1 day of CdCl_2_ treatment. Expression of the *P. alba HMA* gene increased in roots after 1-week and 1-month exposure to CdCl_2_ (Rome et al., 2016). The transcript abundance of *TcNRAMP3*, from the Cd-hyperaccumulator *T.* (*Noccaea*) *caerulescens*, increased steadily up to 96 h after treatment with 500 μM CdCl_2_ (Wei et al., 2009). *CsMTP9* mRNA level increased ∼2.5-fold upon cucumber plant exposure to Cd (Migocka et al., 2015b). However, Cd did not significantly affect *CsMTP1* and *CsMTP4* expression, nor protein levels in cucumber roots (Migocka et al., 2015a). Compared with the low Cd^2+^ concentration treatment, more genes showed decreased expression or no significant difference after high Cd^2+^ concentration treatment. In addition, the maximum expression of those genes, which showed increased Cd-induced expression under 30 μM Cd^2+^ stress, was higher than the corresponding values expressed under 100 μM Cd^2+^ stress, except for genes *MaZIP5* and *MaHMA8*. That observation may be related to the fact that a high Cd concentration is more toxic to plant roots than a low concentration. Maybe different genes function in different tissues, such as the roots. Interestingly, when *MaNRAMP1* (where expression in the root was significantly decreased under Cd^2+^ stress) and *MaZIP4* or *MaHMA3* (where expression in the root was significantly increased under Cd^2+^ stress) were heterologously expressed in yeast, the yeast cells showed considerably increased sensitivity to Cd^2+^.

### The Molecular Mechanism of Transporter Response to Cd^2+^ Stress in Stems

The Cd movement from roots to aerial tissues occurs through transpiration-driven xylem loading, with symplastic and apoplastic transport of free Cd^2+^ or Cd-chelate complexes (Kranner and Colville, 2011; Lux et al., 2011). Several studies have reported that HMA and ZIP family transporters participate in Cd transport across the plasma membrane and into plant shoots (Hanikenne et al., 2008; Wong and Cobbett, 2009). OsZIP3, located in the nodes, is involved in unloading Zn from the xylem into the enlarged vascular bundles of rice, while OsZIP4, 5 and 8 should be responsible for root-to-shoot Zn translocation (Sasaki et al., 2015). *Morus* ZIP1 and 5, which belong to the same group as OsZIP3-5 and 8, may be involved in Cd transport in the stem. Under both 30 and 100 μM Cd^2+^ treatments, *MaZIP1* (5.1- and 1.9-fold increases, respectively) and *MaZIP4* (2.3- and 3.7-fold increases, respectively) showed increased expression. OsHMA2, AtHMA2 and 4 are responsible for Cd translocation to shoots (Wong and Cobbett, 2009; Takahashi et al., 2012; Cun et al., 2014). *Morus* HMA1 and HMA2 belong to the same Group as the above HMAs. Compared with *Morus HMA1*, the expression of *Morus HMA2* was very low in the five different tissues. *MaHMA1* showed no significant difference in expression after 30 μM Cd^2+^ treatment but increased expression (10.8-fold) after 100 μM Cd^2+^ treatment. In addition, *MaHMA8* (5.6- and 10.7-fold increases under low and high Cd^2+^ treatments, respectively) may also play an important role in Cd transport in stem cells. Compared with the low Cd^2+^ concentration treatment, more genes showed increased expression after high Cd^2+^ concentration treatment. This phenomenon may be related to the fact that high Cd concentration is more toxic to plant roots, so that the expression of genes associated with root-to-shoot translocation increased to reduce Cd concentration in the roots. In stems, expression of *PaHMA2* in polar increased after 1-month Cd treatment (Rome et al., 2016). The *PtHMA1* expression level increased in the stem after 1 h Cd treatment (Li et al., 2015). Expression of *OsHMA2* in the roots did not change in the presence of Cd, but expression in the shoots increased in the presence of 1 mM Cd (Takahashi et al., 2012).

### The Molecular Mechanism of Transporter Response to Cd^2+^ Stress in Leaves

The majority of Cd taken up into *Morus* leaves was bound to the cell wall in the leaves (Zhou et al., 2015a). When Cd^2+^ transport into the mesophyll causes inhibition of photosynthesis (Kupper et al., 2007), vacuolar compartmentalization is important in order to reduce Cd-induced toxicity, as it reduces the movement of free Cd into the cytosol (Choppala et al., 2014). In this study, similar numbers of genes showed increased expression under both 30 and 100 μM Cd^2+^ treatments in leaves. Interestingly, *MaIRT1* (20- and 43-fold increases under low and high Cd^2+^ concentrations, respectively), *MaHMA4* (4.2- and 3-fold increases) and *MaNRAMP4* (5.2- and 3.1-fold increases) showed significant increases, and the expression of these genes within their respective families was very high in leaves. The expression level of *PtHMA1* in the leaves gradually increased during exposure to Cd (Li et al., 2015). Growth on elevated Cd concentrations caused the increase of *ZNT1* expression in the spongy mesophyll of young and mature leaves (Kupper and Kochian, 2010). When *MaIRT1* was heterologously expressed in yeast, the cells showed considerably increased sensitivity to Cd^2+^.

Compared to non-accumulator plants, metal hyperaccumulation in hyperaccumulators relies on (at least partly) higher expression (up to 200-fold higher) of various metal transporter genes (Leitenmaier and Kupper, 2013). However, metal transporters show diverse levels of selectivity based on the family to which they belong. The mechanisms of the regulation of expression of the HM transporter genes are largely unknown. There are a few transcription factors known to be involved in Cd stress response, such as CaPF1 (an ERF/AP2-like transcription factor), TaHSFA4a (a member of the heat shock transcription factor family), microRNA268 and OsMYB45 (Tang et al., 2005; Donghwan et al., 2009; Ding et al., 2017; Hu et al., 2017). In our study, several transporter genes were shown to contain MYB binding sites and/or heat stress responsive sites in their promoter sequences. The mechanisms of transcriptional and post-transcriptional gene regulation of HM-specific and non-specific transporters are complex, a topic which needs further functional analysis.

In this study, we identified 31 *Morus* putative transporter genes, analyzed their possible regulation and expression patterns carefully, and performed yeast functional analysis on candidate genes. These results will provide useful information for understanding the complex molecular mechanism of response of mulberry trees under Cd^2+^ stress, which could be exploited in the future for phytoremediation purposes.

## Author Contributions

WF, CL, and AZ conceived and designed the experiments. WF and MQ performed the experiments. WF, CL, BC, and DL analyzed the data. WF, CL, MQ, BC, DL, ZX, and AZ contributed reagents, materials, and analysis tools. WF, CL and AZ wrote the paper. All authors approved the final manuscript.

## Conflict of Interest Statement

The authors declare that the research was conducted in the absence of any commercial or financial relationships that could be construed as a potential conflict of interest.

The reviewer MR-P and handling Editor declared their shared affiliation.

## Footnotes

1 http://pfam.xfam.org/

2 http://www.hmmer.org/

3 https://www.arabidopsis.org/

4 http://rice.plantbiology.msu.edu/index.shtml

5 https://morus.swu.edu.cn/morusdb/

6 http://smart.embl-heidelberg.de/

7 https://www.ncbi.nlm.nih.gov/Structure/cdd/wrpsb.cgi

8 https://wolfpsort.hgc.jp/

9 http://linux1.softberry.com/berry.phtml?topic=protcomppl&group=programs&subgroup=proloc

10 http://www.cbs.dtu.dk/services/TMHMM/

11 http://itol.embl.de/itol.cgi

12 http://gsds.cbi.pku.edu.cn/

13 http://meme-suite.org/tools/meme

14 http://aramemnon.botanik.uni-koeln.de/request.ep

15 http://www.ncbi.nlm.nih.gov/

16 http://bioinformatics.psb.ugent.be/webtools/plantcare/html/

17 https://morus.swu.edu.cn/morusdb/

18 https://cran.r-project.org/web/packages/pheatmap/index.html

19 https://morus.swu.edu.cn/morusdb/transcriptomes

## Supplementary Material

FIGURE S1 Amino acid alignment of ZIP, NRAMP, HMA, and MTP protein.

TABLE S1 The sequences of the primers used in this study.

TABLE S2 The number of ZIP, NRAMP, HMA and MTP transporters in *Arabidopsis*, rice and *Morus*.

TABLE S3 The putative *cis*-elements that are related to stress in the region -1500 upstream of the ATG translation initiation site of four gene families in *M. notabilis.*

TABLE S4 Amino acid sequence identities between the heavy metal transporter of *M. notabilis* and *Ma-GY62*.

DATA SHEET S1 Phylogenetic analyses, classification and functional relatedness of the *NRAMP, HMA*, and *MTP* genes.

DATA SHEET S2 Details of the 20 motifs of the four transporter families.
