# Supplementary material for: Genome-Wide Identification and Characterization of Four Gene Families Putatively Involved in Cadmium Uptake, Translocation and Sequestration in Mulberry
Source: Front Plant Sci. 2018 Jun 29;9:879. doi: 10.3389/fpls.2018.00879 (PMC6034156; doi:10.3389/fpls.2018.00879)
Supplement: FIGURE S1 — Amino acid alignment of ZIP, NRAMP, HMA, and MTP protein. [file Image_1.PDF]

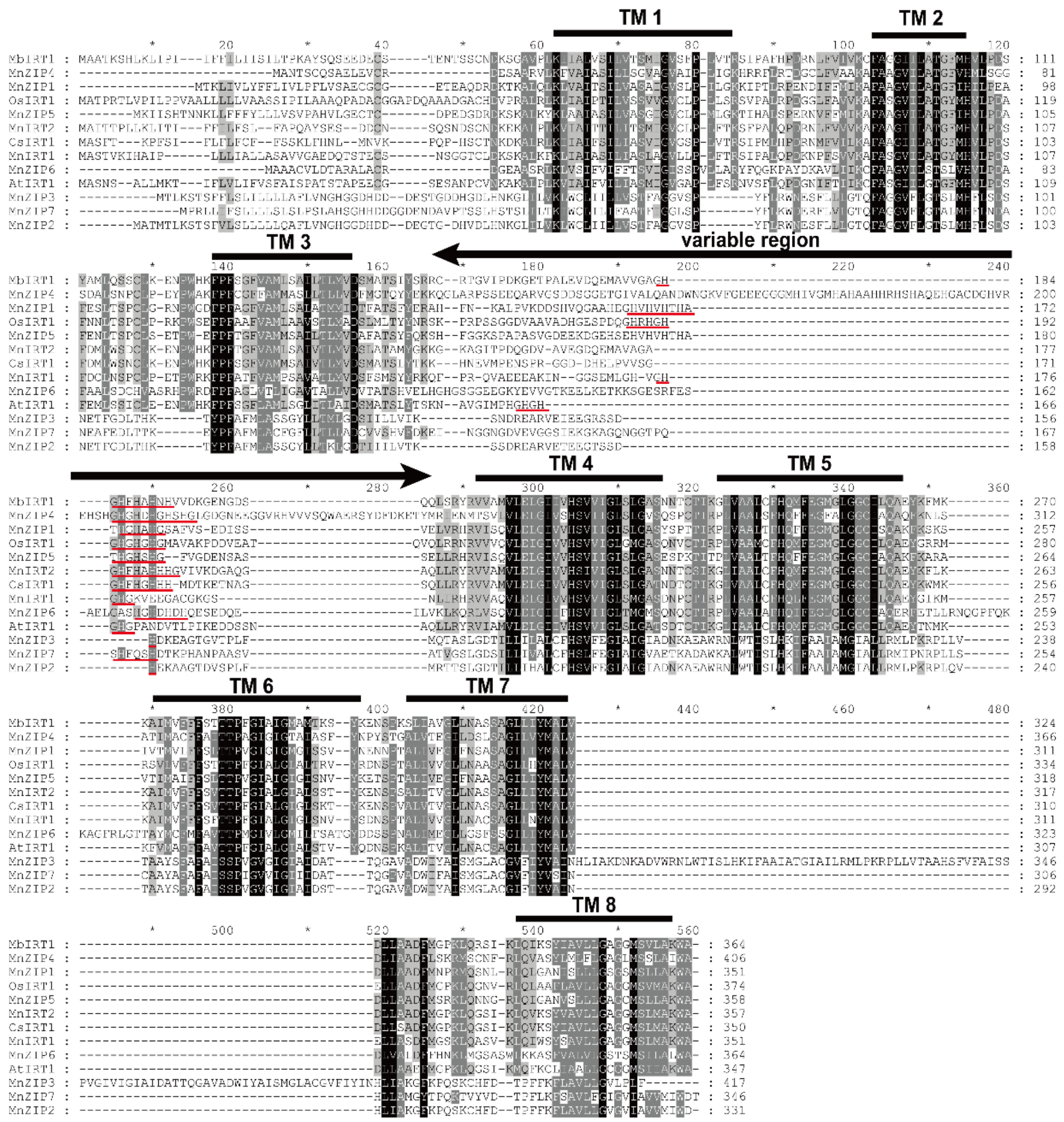

Amino acid alignment of ZIP proteins. Multiple alignment of amino acid sequences of the deduced protein sequences of MnZIPs with AtIRT1, OsIRT1, MbIRT1 and CsIRT1. AtIRT1 (NP_567590) is from *Arabidopsis thaliana*; OsIRT1 (Q75HB1) is from *Oryza sativa*; MbIRT1 (AAO17059) is from *Malus baccata* var. xiaojinensis; and CsIRT1 (ABV72699) is from *Chlamydomonas reinhardtii*. Amino acid residues that are conserved are shown in white type on a black or gray background. Red lines indicate conserved histidine residues.

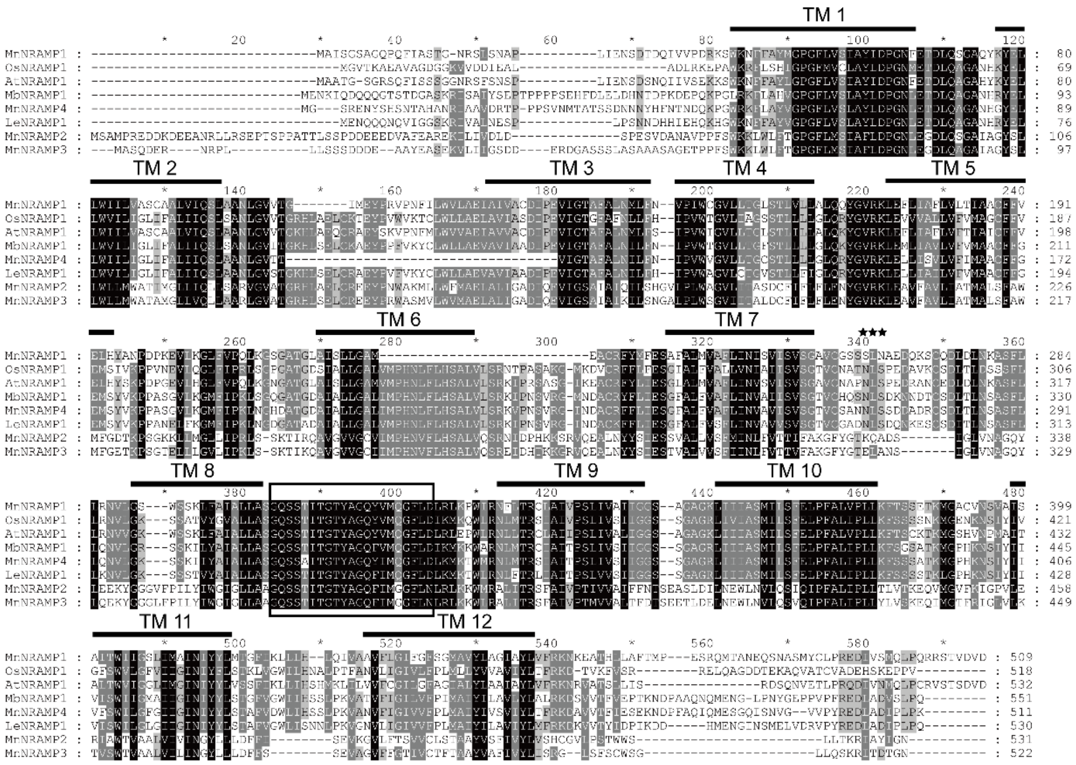

Amino acid alignment of NRAMP proteins. Multiple alignment of amino acid sequences of the deduced protein sequences of MnNRAMPs with OsNRAMP1, AtNRAMP1, MbNRAMP1 and LeNRAMP1. MbNRAMP1 (AAU00158.1) is from *Malus baccata*; AtNRAMP1 (NP_178198.1) is from *Arabidopsis thaliana*; LeNRAMP1 (NP_001234318.1) is from *Solanum lycopersicum*; and OsNRAMP1 (XP_015647629.1) is from *Oryza sativa Japonica Group*. Amino acid residues that are conserved are shown in white type on a black or gray background. The consensus transport motif between TM 8 and 9 is boxed. Red lines indicate conserved histidine residues. The asterisks represent putative N-linked glycosylation sites.

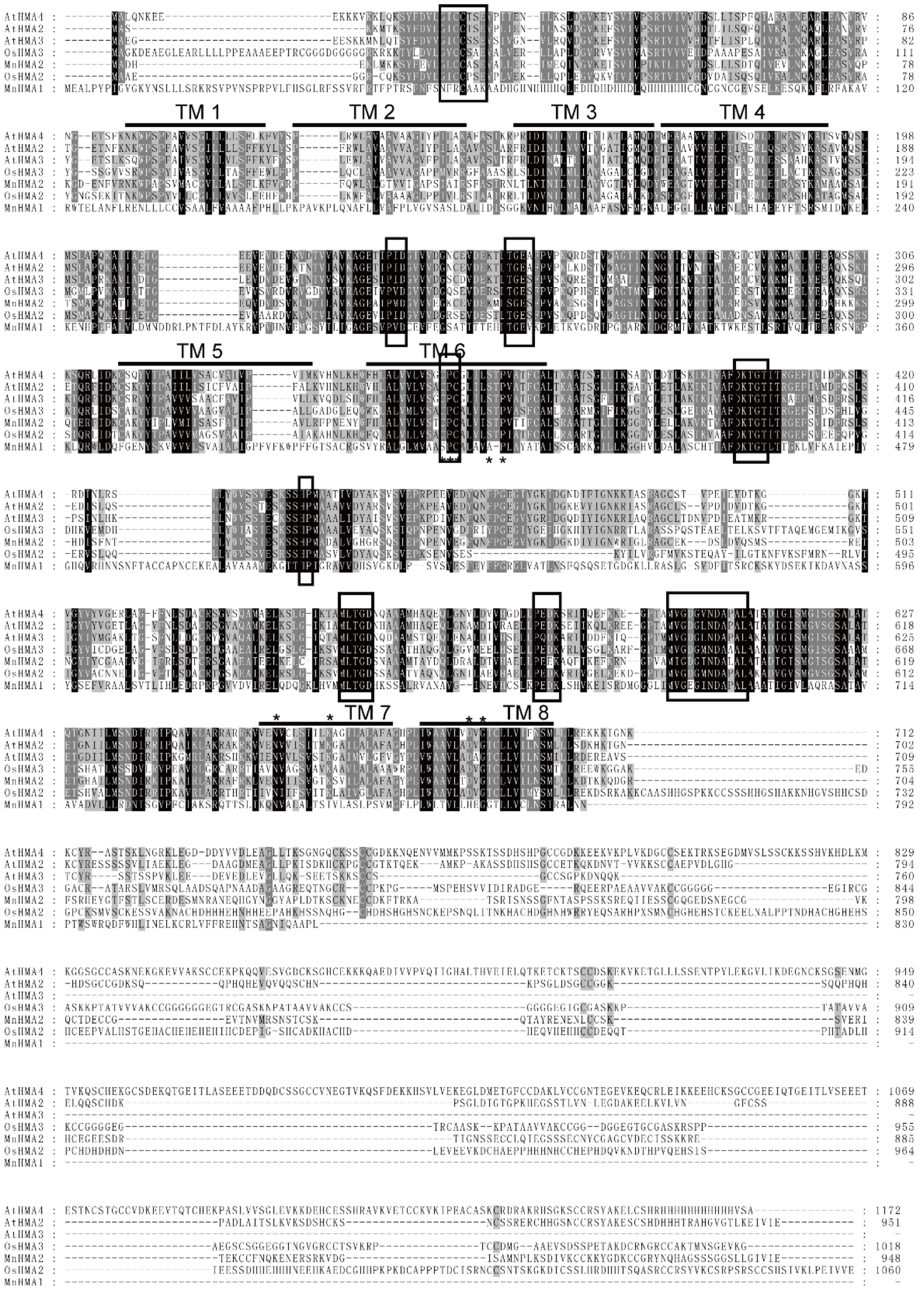

Amino acid alignment of HMA proteins. Multiple alignment of amino acid sequences of the deduced protein sequences of MnHMAs with OsHMA2-3, AtHMA2-4. OsHMA2 (XP_015643659.1) and OsHMA3 (XP_015647368.1) from *Oryza sativa*; AtHMA2 (NP_194740.1), AtHMA3 (NP_194741.2) and AtHMA4 (NP_179501.1) is from *Arabidopsis thaliana*. Amino acid residues that are conserved are shown in white type on a black or gray background. The typical motifs of P1B-ATPases are boxed. The transmembrane domain (TM) of is marked with black lines. The asterisks represent highly conserved residues sites.

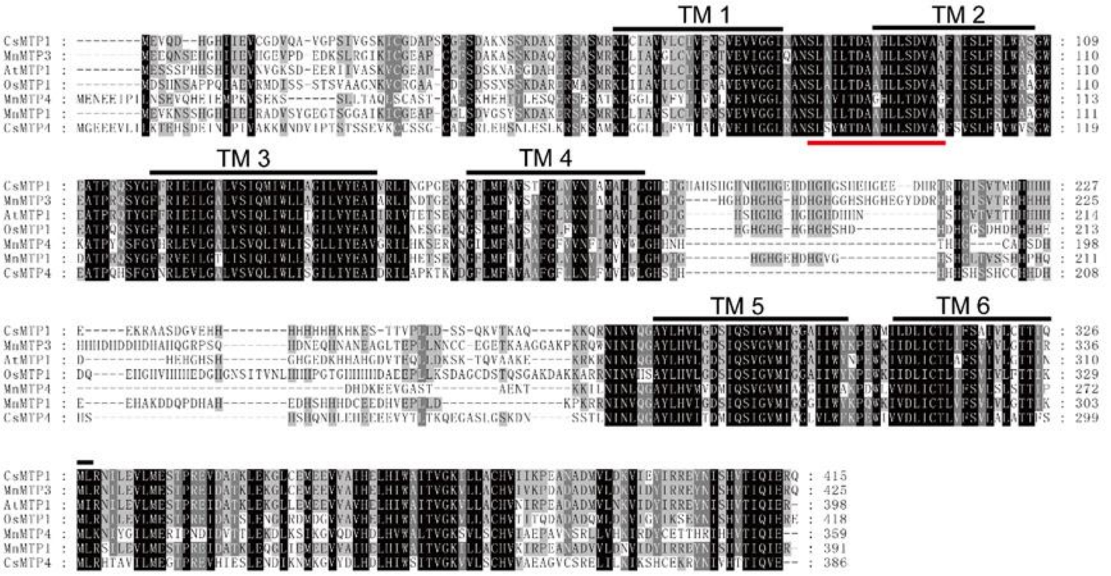

Amino acid alignment of MTP proteins. Multiple alignment of amino acid sequences of the deduced protein sequences of MnMTPs with OsMTP1, CsMTP1, 4. OsMTP1 (XP_015640283.1) from *Oryza sativa*; CsMTP1 (XP_004145030.1) and CsMTP4 (NP_001295861.1). Amino acid residues that are conserved are shown in white type on a black or gray background. The consensus transport motif is marked with red lines. The transmembrane domain (TM) are marked with black lines.
