# Supplementary material for: Genome-Wide Identification and Characterization of Four Gene Families Putatively Involved in Cadmium Uptake, Translocation and Sequestration in Mulberry
Source: Front Plant Sci. 2018 Jun 29;9:879. doi: 10.3389/fpls.2018.00879 (PMC6034156; doi:10.3389/fpls.2018.00879)
Supplement: TABLE S1 — The sequences of the primers used in this study. [file Table_1.DOCX]

**S1 Table** The sequences of the primers used in this study

| Primer | Sequence (5’-3’) | |
| --- | --- | --- |
| MaIRT1 | F:5’-ATGGCGTCTACCGTTAAAATCC-3’ | |
|  | R:5’-CTAAGCCCACTTTGCCAT-3’ | |
| MaIRT2 | F:5’- ATGGCCATCACTACACCACTTC-3’ | |
|  | R:5’- TCAAGCCCATTTTGCCAT-3’ | |
| MaZIP1 | F:5’- ATGACGAAGCTAATAGTAC-3’ | |
|  | R:5’- TTAAGCCCATTTGGCCAAT-3’ | |
| MaZIP2 | F:5’- ATGGCCACCATGACTCTC -3’ | |
|  | R:5’- TTAATCCCATATCATAACAACG-3’ | |
| MaZIP3 | F:5’- ATGACTCTCAAGTCAACCTCT -3’ | |
|  | R:5’- TCAAAACAACGGCAATAC -3’ | |
| MaZIP4 | F:5’-ATGGCGAATACAAGTTGCCAGAG -3’ | |
|  | R;5’-TCAAGCCCAAATAGCTAATG-3’ | |
| MaZIP5 | F:5’-ATGAAGATAATTTCTCACAC-3’ | |
|  | R;5’-TTAAGCCCATTTAGCCAAAAGAG-3’ | |
| MaZIP6 | F:5’-ATGGCTGCAGCGTGCGTCCTTG-3’ | |
|  | R:5’-TTAAGCCCAAAGAGCTAG-3’ | |
| MaZIP7 | F:5’-ATGCCTCGTCTCCTCATTTTCTC-3’ | |
|  | R:5’-TCAAGTGTCCCAGATCATCACAAC-3’ | |
| MaNRAMP1 | F:5’-ATGGCAATATCCGGTTCG-3’ | |
|  | R:5’-TTAGTCAACATCAACAGTGGACC-3’ | |
| MaNRAMP2 | F: 5’-ATGAGCGCTATGCCAAGGGAAG-3’ | |
|  | R: 5’-TTAGTTGCCGATGTATGC-3’ | |
| MaNRAMP3 | F: 5’-ATGGCGTCCCAAGACGAACGCAAC-3’ | |
|  | R: 5’-TCAGTTTCCTGTGTCTGTGATCC-3’ | |
| MaNRAMP4 | F: 5’-ATGGGAAGCCGAGAAAAC-3’ | |
|  | R: 5’-CTACTTAGGTAAAGGAATATCGGC-3’ | |
| MaHMA1 | F: 5’-ATGGAAGCTCTTCCATATCCGATC-3’ | |
|  | R: 5’-TCACAAAGGAGCAGCCTGGATATTG-3’ | |
| MaHMA2 | F: 5’-ATGGCTGATCATGAGAAACT-3’ | |
|  | R: 5’-CTATTCAATAACAATACCCAACAAG-3’ | |
| MaHMA3 | F: 5’-ATGGCAGCGAAGCTTTTGGCGTTG-3’ | |
|  | R: 5’-CTACTCAATGCTTATTCCACG-3’ | |
| MaHMA4 | F: 5’-ATGGAAGCCAATGGAAACGATGAC-3’ | |
|  | R: 5’-TCAAGTGTGTTCAACTTGCAAAG-3’ | |
| MaHMA5 | F: 5’-ATGGCTTCAAAGTATGTTCCTTGG-3’ | |
|  | R: 5’-TTACTCAACCATTACTCCTCGTATC-3’ | |
| MaHMA6 | F: 5’-ATGGAGTCCACACTATCCTCCGTC-3’ | |
|  | R: 5’-TCATGCACCTTTCCATCTAGCATC-3’ | |
| MaHMA7 | F: 5’-ATGGCGCCGAACAGCAGAAGCCTC-3’ | |
|  | R: 5’-CTATTCTACAGTTATTTCTAGTATAG-3’ | |
| MaHMA8 | F: 5’-ATGACTACTGGTTTTCTGACCATTTC-3’ | |
|  | R: 5’-CTAACTCCTCTTGTCAGACCCATGG-3’ | |
| MaMTP1 | F: 5’-ATGGAAGTGAAAAATTCTTCAC-3’ | |
|  | R: 5’-CTAACGCTCAATTTGTATA-3’ | |
| MaMTP2 | F: 5’- ATGGGATTCAGATTCCACAAT-3’ | |
|  | R: 5’- CTAACTTCCCACGTCAGCTT-3’ | |
| MaMTP3 | F: 5’-ATGGAAGAGCAAAACTCTG-3’ | |
|  | R: 5’-GTTTAACTCGCAGTCATC-3’ | |
| MaMTP4 | F: 5’-ATGGAAAATGAAGAAATTCCCAT-3’ | |
|  | R: 5’-TACTCGATTTGTATTGTTACAT-3’ | |
| MaMTP5 | F: 5’-ATGGCGGAGCCGATGGGTCTGG-3’ | |
|  | R: 5’-CTAAGAGTGTGCCTGAGCATGC-3’ | |
| MaMTP6 | F: 5’-ATGGAGGGAAATTCGGAGGTGAG-3’ | |
|  | R: 5’-TCAAGGCTGAGAATTTGGCAGT-3’ | |
| MaMTP7 | F: 5’-ATGCCTGTGCCGAGGAAAGC-3’ | |
|  | R: 5’-TCATGGGAACAAGTCCGTTG-3’ | |
| MaMTP8 | F: 5’-ATGGAAGGTGATTTGGATTA-3’ | |
|  | R: 5’-TTAAGGCTGGCTATTGGGGAGT-3’ | |
| MaMTP9 | F: 5’-ATGGTAAAAGCTACCAACG-3’ | |
|  | R: 5’-TCATGGGACTAATTTTGG-3’ | |
| MaMTP10 | F: 5’-ATGGAGAACATCGGCGGCGGAAG-3’ | |
|  | R: 5’-TCAGGCCTTTCGCTTGTGCTCC-3’ | |
| qMaIRT1 | F:5’-GACAGGTAGCCGAAGAGGAG-3’ | |
|  | R:5’-AATACCTGAGCGACAACACG-3’ | |
| qMaIRT2 | F:5’- CGATTGTAATCGGCCTTTCT-3’ | |
|  | R:5’- TGCCTGTAGAATGCAACCTC-3’ | |
| qMaZIP1 | F:5’-TTTCAAGTCTCTGCCGAATG-3’ | |
|  | R:5’-GCGCTGGCTACAAGTATTGA-3’ | |
| qMaZIP2 | F:5’-TGCACAACAAAGGGTTGATT-3’ | |
|  | R:5’-AGCGGAGGAAGTATGGTGAC-3’ | |
| qMaZIP3 | F:5’-TAACAAGGCGGATGTATGGA-3’ | |
|  | R:5’-GTAGCATCCTCAGAATCGCA-3’ | |
| qMaZIP4 | F:5’-ACGCTGCTCACCATAGACAC-3’ | |
|  | R;5’-CCTAACACCACCTTCCTCGT-3’ | |
| qMaZIP5 | F:5’-CAGCATCAGCTGGGATCTTA-3’ | |
|  | R;5’-CCTATTTGGAGCCTTGCATT-3’ | |
| qMaZIP6 | F:5’-GACGCTGATCGGAGTAGTCA-3’ | |
|  | R:5’-CTCCTTCAACTCCTCCTTCG-3’ | |
| qMaZIP7 | F:5’-CTCCACTCCACGTCCCTAAT-3’ | |
|  | R:5’-AACCTCTCGTCCCATTTCAG-3’ | |
| qMaNRAMP1 | F:5’-CATCAGAGAGGCATGCAGAT -3’ | |
|  | R:5’- CAGACTTGAAGAACCGCAAA-3’ | |
| qMaNRAMP2 | F:5’-TGGTTCTGTTGTCTGCATCA-3’ | |
|  | R;5’-TTAGCAAGCTCCACCAAGTG-3’ | |
| qMaNRAMP3 | F:5’-GTGGCTGCCCTGGTAATACT-3’ | |
|  | R:5’-CGGGAGATGAGGTAAACGAT-3’ | |
| qMaNRAMP4 | F:5’-TATGCAAGGCTGAATACCCA-3’ | |
|  | R:5’-ATTGTGCGGCATGATAAGAG-3’ | |
| qMaHMA1 | F:5’-CGCTGTCAATGCATCTTCTT -3’ | |
|  | R;5’-TGACATCCACAACTCCAGGT -3’ | |
| qMaHMA2 | F:5’-AGCAACACGGATACAATGGA-3’ | |
|  | R:5’-AGAACTTGGGCTCGCTGTAT-3’ | |
| qMaHMA3 | F:5’-CAGCTTGCTCTCCAGTTTGGG-3’ | |
|  | R:5’-CGCGCCTACTCCAGTACCAA-3’ | |
| qMaHMA4 | F:5’-GTTGGGAAGCCATTGGTAGT-3’ | |
|  | R;5’-TCACTGTTGGCCTCTGTAGC-3’ | |
| qMaHMA5 | F:5’-ATAGCTTGCCCTTGTGCTTT-3’ | |
|  | R:5’-TGCACTTTCTAATGCTTGGC-3’ | |
| qMaHMA6 | F:5’-TATTGTGGAAGCTGCTCAGG-3’ | |
|  | R:5’-CTTTGAACCCAGTCCAAGGT-3’ | |
| qMaHMA7 | F:5’-AGAAAGTTGGCTCCCAGAAA-3’ | |
|  | R;5’-TTAGCACCAACTCCTGTTGC-3’ | |
| qMaHMA8 | F:5’-GCCGAAGTAGATGGGTGTTT-3’ | |
|  | R:5’-ACGATTGGTGAATTGCATGT-3’ | |
| qMaMTP1 | F:5’-GCTTGCTGGGATATTGGTTT-3’ | |
|  | R:5’-TCATGGCCTAGCAATAGCAC-3’ | |
| qMaMTP2 | F:5’-GTGGCATTTGGGCCATTCCC-3’ | |
|  | R;5’-CGCGAGACCTAGCCGGAATA-3’ | |
| qMaMTP3 | F:5’-CCAGACATCACCATCACCAT-3’ | |
|  | R:5’-CAGCTTTCGTTTCACCTTCA-3’ | |
| qMaMTP4 | F:5’-TGCAGCTCATTTGGCTAATC-3’ | |
|  | R:5’-CCCATTGACCCTTTCACTCT-3’ | |
| qMaMTP5 | F:5’-TCTCTTGCGACAGCCCAACA-3’ | |
|  | R;5’-GCAATCATGGAGGCCACGAG-3’ | |
| qMaMTP6 | F:5’-GTGGGATTAGTCGCGGCTGT-3’ | |
|  | R:5’-GGGCAGATTGTCCAACGAGTG-3’ | |
| qMaMTP7 | F:5’-TACCGGTCTTGCTATTGCTG -3’ | |
|  | R:5’-ATAGCGACCATTCCAAGGAG-3’ | |
| qMaMTP8 | F:5’-GGGCATTGTCATATTTGCAG-3’ | |
|  | R;5’-GAACAATTGGTCCGAAGTCA-3’ | |
| qMaMTP9 | F:5’-ACCTGAGAGGGACCCAGTGA-3’ | |
|  | R:5’-CCGCAGCTGCAAGTCCAAT-3’ | |
| qMaMTP10 | F:5’-GCCGACGACTGCAACAGAAG-3’ | |
|  | R:5’-GAGGTCGAGCCTCCACGAAA-3’ | |
| qMaACTIN3 | F:5’-GCATGAAGATCAAGGTGGTG-3’ | |
|  | R:5’-CATCTGCTGGAAGGTGCTAA-3’ | |
| pYPGE15-IRT1 | F: 5’-GCCTCTAGAATGGCGTCTACCGTTAAAATCC-3’ | Xba I |
|  | R: 5’-GCCGGTACCCTAAGCCCACTTTGCCAT-3’ | Kpn I |
| pYPGE15-NRAMP1 | F:5’-GCCTCTAGAATGGCAATATCCGGTTCG-3’ | Xba I |
|  | R: 5’-GCCGGTACCTTAGTCAACATCAACAGTGGACC-3’ | Kpn I |
| pYPGE15-ZIP4 | F:5’-GCCTCTAGAATGGCGAATACAAGTTGCCAGAG-3’ | Xba I |
|  | R: 5’-GCCGGTACCTCAAGCCCAAATAGCTAATG-3’ | Kpn I |
| pYPGE15-HMA3 | F:5’-AACTGCAGATGGCAGCGAAGCTTTTG-3’ | Pst I |
|  | R: 5’-CCGCTCGAGCTACTCAATGCTTATTCCACG-3’ | Xho I |
