# Supplementary material for: Genome-Wide Identification and Characterization of Four Gene Families Putatively Involved in Cadmium Uptake, Translocation and Sequestration in Mulberry
Source: Front Plant Sci. 2018 Jun 29;9:879. doi: 10.3389/fpls.2018.00879 (PMC6034156; doi:10.3389/fpls.2018.00879)
Supplement: TABLE S2 — The number of ZIP, NRAMP, HMA and MTP transporters in Arabidopsis, rice and Morus. [file Table_2.docx]

**Table S2 the number of ZIP, NRAMP, HMA and MTP transporters in *Arabidopsis*, *rice* and *Morus*.**

| Species | ZIP | NRAMP | HMA | MTP |
| --- | --- | --- | --- | --- |
| *A. thaliana* | 15 | 6 | 8 | 12 |
| *O. sativa* | 11 | 7 | 9 | 10 |
| *M. notabilis* | 9 | 4 | 8 | 10 |
