# Supplementary material for: Genome-Wide Identification and Characterization of Four Gene Families Putatively Involved in Cadmium Uptake, Translocation and Sequestration in Mulberry
Source: Front Plant Sci. 2018 Jun 29;9:879. doi: 10.3389/fpls.2018.00879 (PMC6034156; doi:10.3389/fpls.2018.00879)
Supplement: TABLE S3 — The putative cis-elements that are related to stress in the region -1500 upstream of the ATG translation initiation site of four gene families in M. notabilis. [file Table_3.docx]

**S3 Table** The putative *cis*-elements that are related to stress in the region -1500 upstream of the ATG translation initiation site of four gene families in *M. notabilis*.

| **Gene name** | ***cis*-elements related to abiotic stress^a^** |
| --- | --- |
| *MnIRT1* | ABRE ARE HSE LTR TC-rich repeats TCA-element WUN-motif |
| *MnIRT2* | ARE CGTCA-motif HSE MBS TC-rich repeats TGACG-motif |
| *MnZIP1* | ARE HSE MBS TC-rich repeats TCA-element WUN-motif |
| *MnZIP2* | LTR TC-rich repeats |
| *MnZIP3* | ARE LTR MBS TC-rich repeats TCA-element W box |
| *MnZIP4* | ABRE CGTCA-motif MBS TCA-element TGA-element TGACG-motif WUN-motif W box |
| *MnZIP5* | ABRE ARE GARE-motif HSE MBS TC-rich repeats TCA-element TGACG-motif |
| *MnZIP6* | HSE MBS TCA-element WUN-motif W box |
| *MnZIP7* | ARE HSE MBS TC-rich repeats |
| *MnNRAMP1* | ARE CGTCA-motif ERE MBS TC-rich repeats TGACG-motif W box |
| *MnNRAMP2* | ABRE ARE CGTCA-motif GARE-motif MBS TC-rich repeats TGACG-motif |
| *MnNRAMP3* | ARE GARE-motif HSE LTR MBS TC-rich repeats TCA-element |
| *MnNRAMP4* | ABRE ARE CGTCA-motif HSE MBS TC-rich repeats TCA-element TGACG-motif |
| *MnHMA1* | ABRE HSE LTR MBS TC-rich repeats |
| *MnHMA2* | ABRE ARE CGTCA-motif HSE LTR TC-rich repeats TCA-element TGACG-motif |
| *MnHMA3* | ABRE ARE ERE GARE-motif HSE MBS TC-rich repeats TCA-element TGA-element W box |
| *MnHMA4* | ABRE ARE CGTCA-motif GARE-motif HSE MBS TCA-element TGA-element TGACG-motif |
| *MnHMA5* | ARE LTR TC-rich repeats TCA-element TGA-element WUN-motif |
| *MnHMA6* | ABRE ARE ERE HSE TCA-element TGA-element |
| *MnHMA7* | ABRE ARE GARE-motif HSE MBS W box |
| *MnHMA8* | ABRE ARE CGTCA-motif GARE-motif HSE MBS TCA-element TGACG-motif |
| *MnMTP1* | ARE TCA-element W box |
| *MnMTP2* | ARE CGTCA-motif ERE HSE MBS TC-rich repeats TCA-element TGACG-motif W box WUN-motif |
| *MnMTP3* | CGTCA-motif HSE MBS TC-rich repeats TCA-element TGACG-motif |
| *MnMTP4* | ARE CGTCA-motif HSE TC-rich repeats TCA-element TGACG-motif W box |
| *MnMTP5* | ARE GARE-motif HSE LTR TC-rich repeats |
| *MnMTP6* | ABRE CGTCA-motif MBS TC-rich repeats TCA-element TGACG-motif W box |
| *MnMTP7* | ABRE GARE-motif HSE MBS TC-rich repeats TCA-element |
| *MnMTP8* | ARE CGTCA-motif GARE-motif HSE MBS TC-rich repeats TCA-element TGA-element TGACG-motif |
| *MnMTP9* | ABRE ARE HSE TCA-element |
| *MnMTP10* | ABRE ARE GARE-motif HSE MBS TC-rich repeats TCA-element TGA-element |

^a^ Abbreviations for the *cis*-element used are as follows:

| **Motif** | **function** |
| --- | --- |
| ABRE | cis-acting element involved in the abscisic acid responsiveness |
| ARE | cis-acting regulatory element essential for the anaerobic induction |
| HSE | cis-acting element involved in heat stress responsiveness |
| LTR | cis-acting element involved in low-temperature responsiveness |
| WUN-motif | wound-responsive element |
| TC-rich repeats | cis-acting element involved in defense and stress responsiveness |
| TCA-element | cis-acting element involved in salicylic acid responsiveness |
| CGTCA-motif | cis-acting regulatory element involved in the MeJA-responsiveness |
| MBS | MYB binding site involved in drought-inducibility |
| TGACG-motif | cis-acting regulatory element involved in the MeJA-responsiveness |
| ERE | ethylene-responsive element |
| TGA-element | auxin-responsive element |
| GARE-motif | gibberellin-responsive element |
| W box | wounding and pathogen respons |
