# Supplementary material for: Genome-Wide Identification and Characterization of Four Gene Families Putatively Involved in Cadmium Uptake, Translocation and Sequestration in Mulberry
Source: Front Plant Sci. 2018 Jun 29;9:879. doi: 10.3389/fpls.2018.00879 (PMC6034156; doi:10.3389/fpls.2018.00879)
Supplement: TABLE S4 — Amino acid sequence identities between the heavy metal transporter of M. notabilis and Ma-GY62. [file Table_4.docx]

**S4 Table** Amino acid sequence identities between the heavy metal transporter of *M. notabilis* and *Ma-GY62*

| **Gene name^a^** | **Amino acid sequence identity** | **Gene name** | **Amino acid sequence identity** | **Gene name** | **Amino acid sequence identity** | **Gene name** | **Amino acid sequence identity** |
| --- | --- | --- | --- | --- | --- | --- | --- |
| *MnIRT1*/*MaIRT1* | 98.7% | *MnNRAMP1/MaNRAMP1* | 93% | *MnHMA1/MaHMA1* | 97% | *MnMTP1/MaMTP1* | 99% |
| *MnIRT2/MaIRT2* | 97% | *MnNRAMP2/MaNRAMP2* | 98% | *MnHMA2/MaHMA2* | 95% | *MnMTP2/MaMTP2* | 99% |
| *MnZIP1/MaZIP1* | 98% | *MnNRAMP3/MaNRMP3* | 99% | *MnHMA3/MaHMA3* | 94% | *MnMTP3/MaMTP3* | 96.7% |
| *MnZIP2/MaZIP2* | 92% | *MnNRAMP4/MaNRAMP4* | 89% | *MnHMA4/MaHMA4* | 99% | *MnMTP4/MaMTP4* | 97% |
| *MnZIP3/MaZIP3* | 100% |  |  | *MnHMA5/MaHMA5* | 98% | *MnMTP5/MaMTP5* | 99% |
| *MnZIP4/MaZIP4* | 92% |  |  | *MnHMA6/MaHMA6* | 96% | *MnMTP6/MaMTP6* | 98% |
| *MnZIP5/MaZIP5* | 98% |  |  | *MnHMA7/MaHMA7* | 98% | *MnMTP7/MaMTP7* | 96% |
| *MnZIP6/MaZIP6* | 93% |  |  | *MnHMA8/MaHMA8* | 98% | *MnMTP8/MaMTP8* | 98% |
| *MnZIP7/MaZIP7* | 98% |  |  |  |  | *MnMTP9/MaMTP9* | 87% |
|  |  |  |  |  |  | *MnMTP10/MaMTP10* | 97% |

**GeneBank accession numbers：MaIRT1 (MG773163), MaIRT2 (MG773162), MaZIP1 (MG773164), MaZIP2 (MG773170), MaZIP3 (MG773169), MaZIP4 (MG773166), MaZIP5 (MG773165), MaZIP6 (MG773167), MaZIP7(MG773168), MaNRAMP1 (MG773172), MaNRAMP2(MG773171), MaNRAMP3 (MG773173), MaNRAMP4 (MG773174), MaHMA1 (MG773177), MaHMA2 (MG773181), MaHMA3 (MG773179), MaHMA4 (MG773182), MaHMA5 (MG773178), MaHMA6 (MG773176), MaHMA7 (MG773180), MaHMA8 (MG773175), MaMTP1 (MG773183), MaMTP2 (MG773191), MaMTP3 (MG773189), MaMTP4 (MG773185), MaMTP5 (MG773186), MaMTP6 (MG773184), MaMTP7 (MG773192), MaMTP8 (MG773187), MaMTP9 (MG773188), and MaMTP10 (MG773190).**
