# Supplementary material for: Genome-Wide Identification and Characterization of Four Gene Families Putatively Involved in Cadmium Uptake, Translocation and Sequestration in Mulberry
Source: Front Plant Sci. 2018 Jun 29;9:879. doi: 10.3389/fpls.2018.00879 (PMC6034156; doi:10.3389/fpls.2018.00879)
Supplement: DATA SHEET S1 — Phylogenetic analyses, classification and functional relatedness of the NRAMP, HMA, and MTP genes. [file Data_Sheet_1.ZIP › additional file 1 (Phylogenetic relationships)/S Frigure2.docx]

*NRAMP*


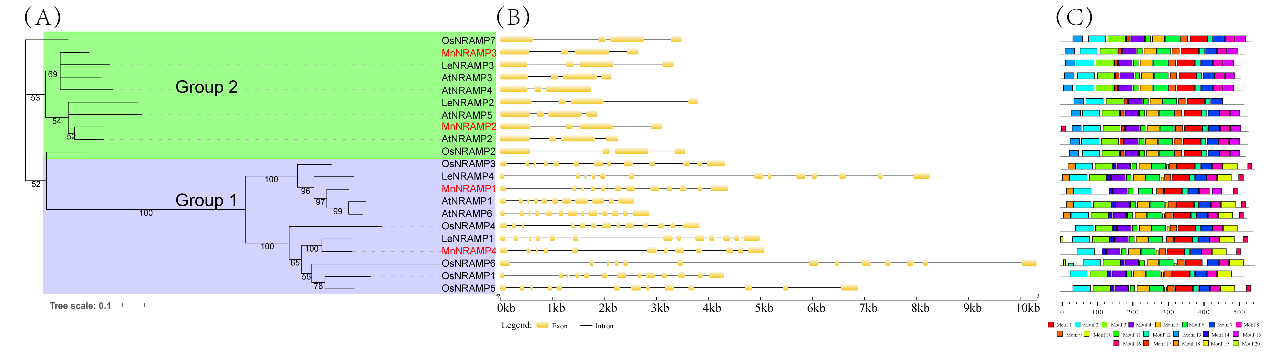


*HMA*

*
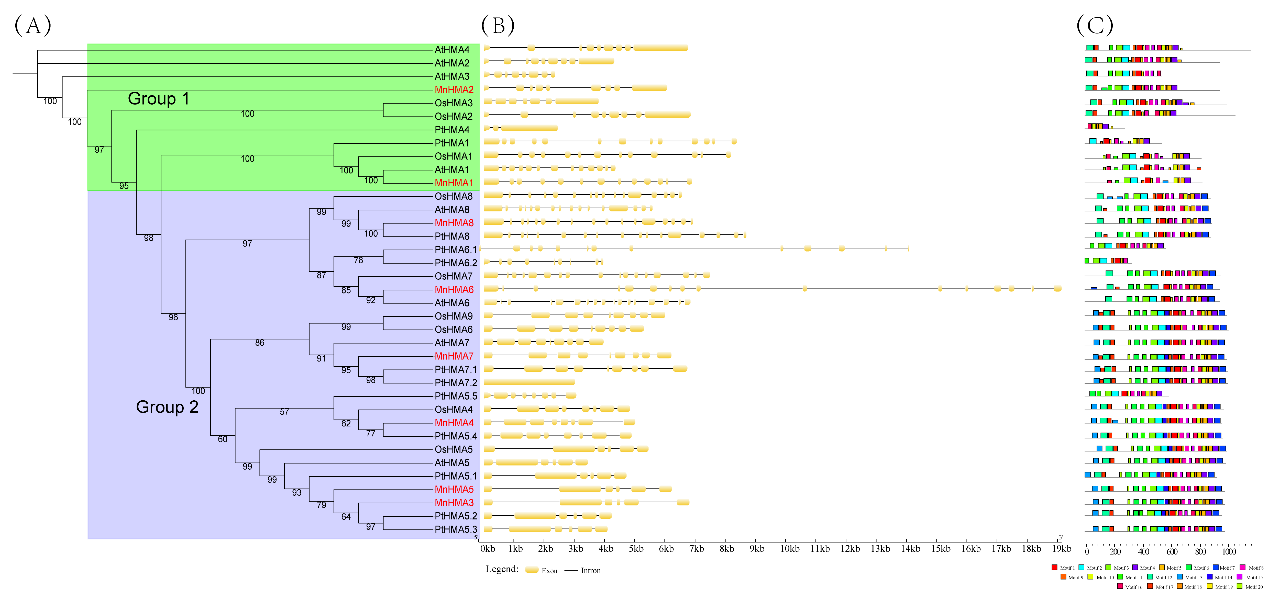
*

*MTP*


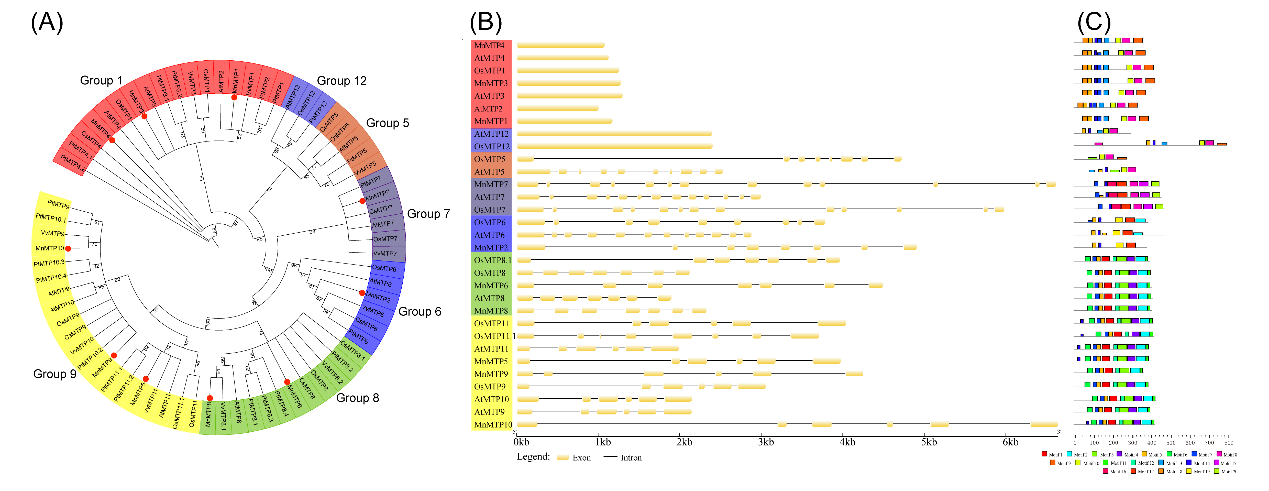


**Supplemental Figure 2. Phylogenetic relationships, gene structure and motif composition of NRAMP, HMA and MTP genes in *Arabidopsis* (At), rice (Os), *Morus* (Mn) and other species. (A)** Multiple sequence alignments of the full-length protein sequences of NRAMP, HMA and MTP genes were performed using the ClustalX version 2.1. Phylogenetic trees were performed with a maximum likelihood method using TREE-PUZZLE 5.3. rc16. The number of puzzling steps was set 10000 times. **(B)** Exon/intron structures. Yellow boxes and black lines, respectively represent exons and introns. **(C)** Schematic representation of the conserved motifs elucidated by MEME 4.11.2. A number in the colored box represents each motif, and the black lines represent the Non-conserved sequences.
