# Supplementary figures and images for: Genome-Wide Identification and Characterization of Four Gene Families Putatively Involved in Cadmium Uptake, Translocation and Sequestration in Mulberry
Source: Front Plant Sci. 2018 Jun 29;9:879. doi: 10.3389/fpls.2018.00879 (PMC6034156; doi:10.3389/fpls.2018.00879)

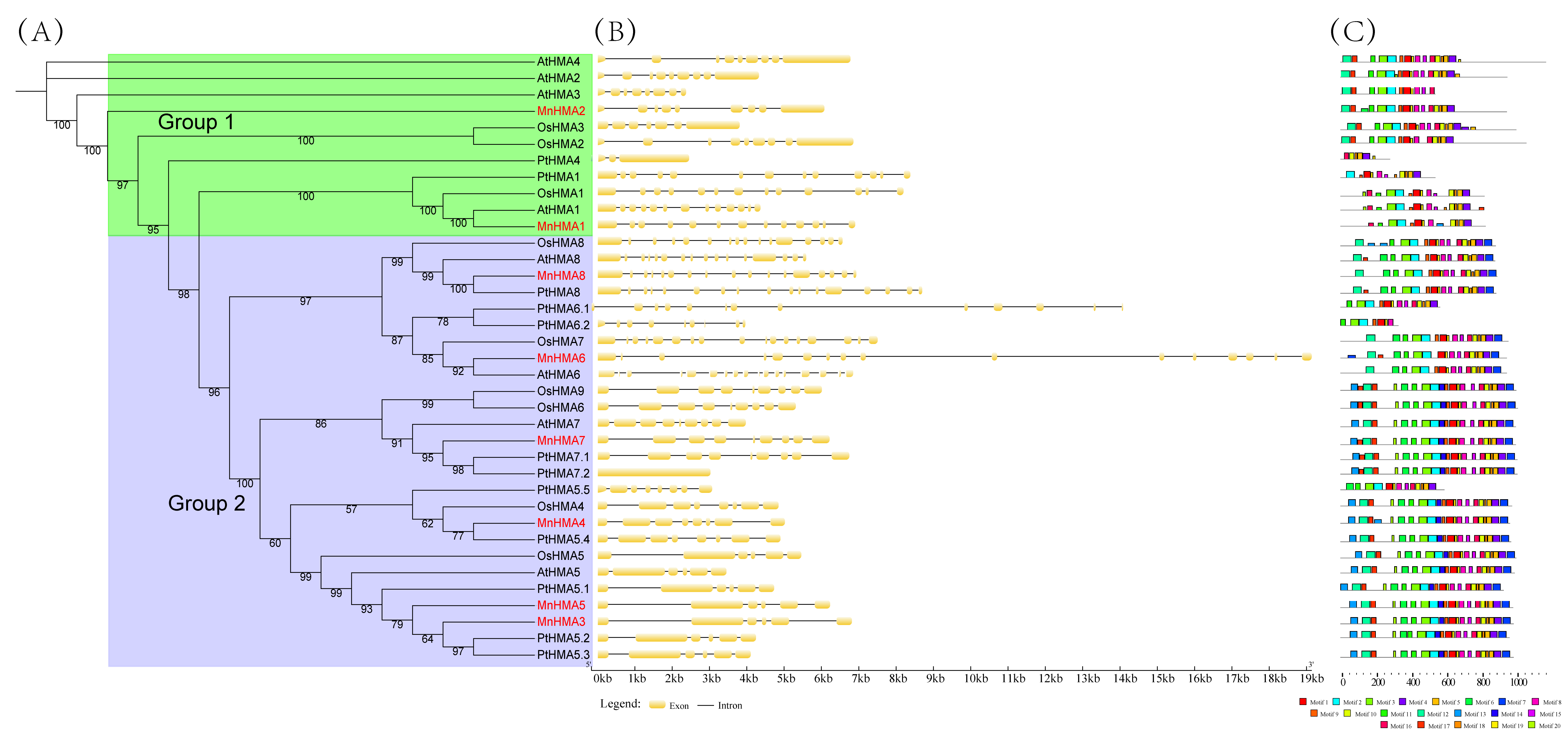

Supplement: DATA SHEET S1 — Phylogenetic analyses, classification and functional relatedness of the NRAMP, HMA, and MTP genes. [file Data_Sheet_1.ZIP › additional file 1 (Phylogenetic relationships)/HMAtree-01.tif]

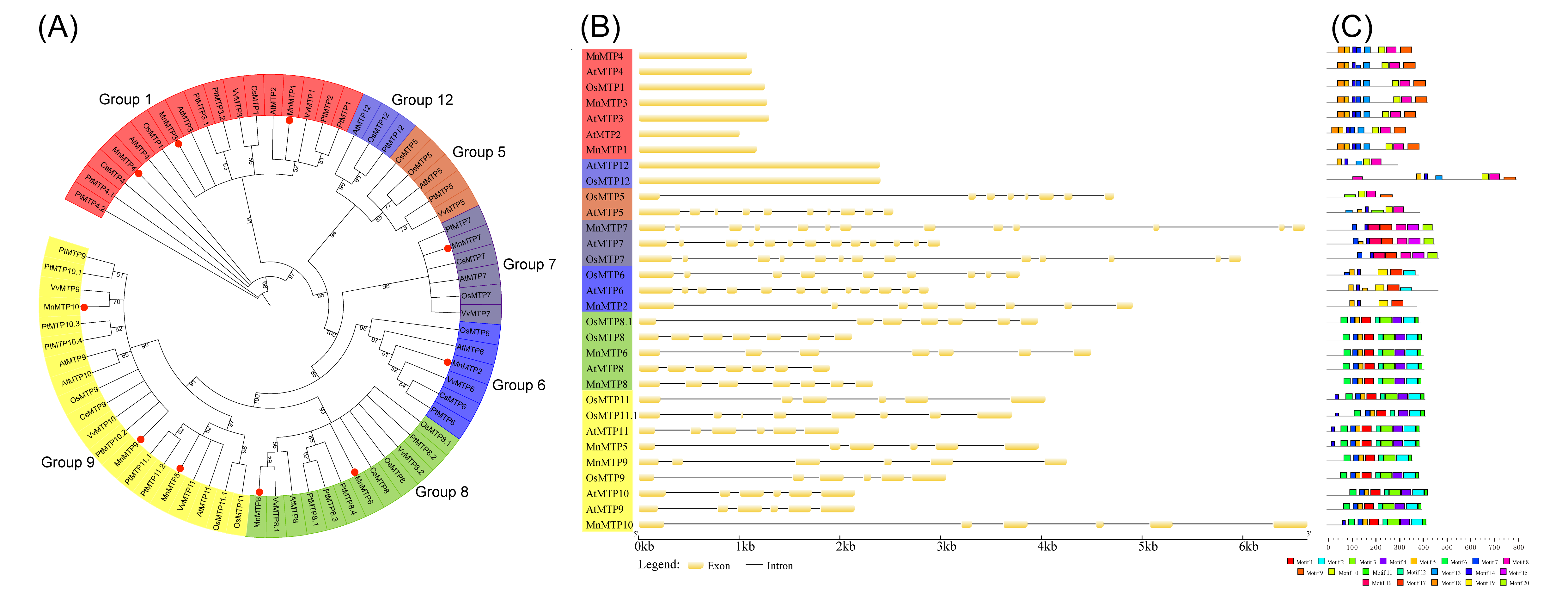

Supplement: DATA SHEET S1 — Phylogenetic analyses, classification and functional relatedness of the NRAMP, HMA, and MTP genes. [file Data_Sheet_1.ZIP › additional file 1 (Phylogenetic relationships)/MTP-01.tif]

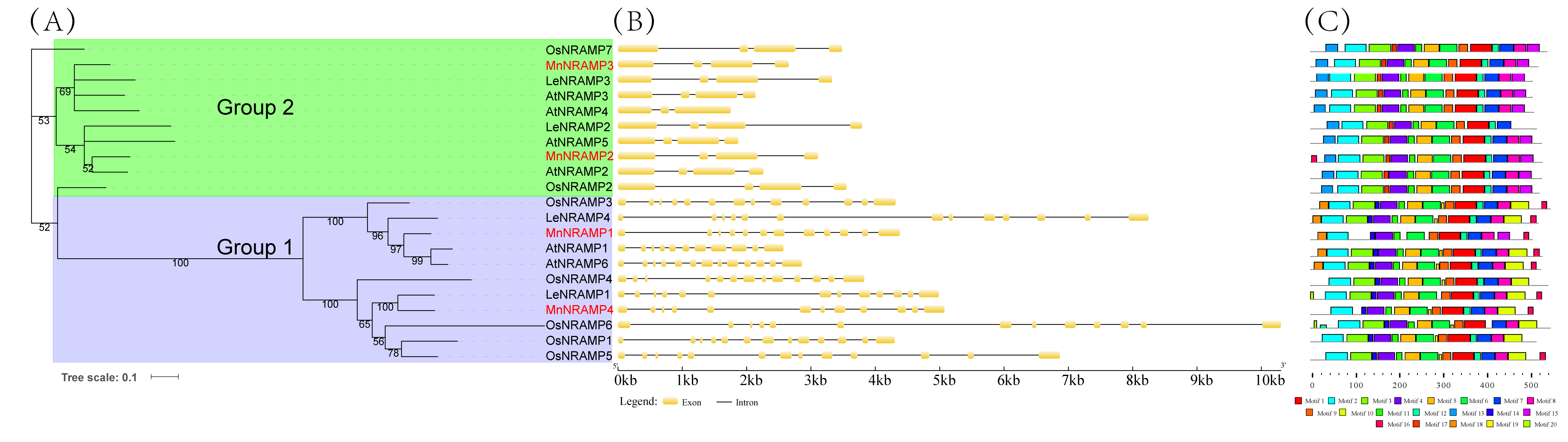

Supplement: DATA SHEET S1 — Phylogenetic analyses, classification and functional relatedness of the NRAMP, HMA, and MTP genes. [file Data_Sheet_1.ZIP › additional file 1 (Phylogenetic relationships)/NRAMPtree-01.tif]
