# Supplementary material for: Genome-Wide Identification and Characterization of Four Gene Families Putatively Involved in Cadmium Uptake, Translocation and Sequestration in Mulberry
Source: Front Plant Sci. 2018 Jun 29;9:879. doi: 10.3389/fpls.2018.00879 (PMC6034156; doi:10.3389/fpls.2018.00879)
Supplement: DATA SHEET S2 — Details of the 20 motifs of the four transporter families. [file Data_Sheet_2.ZIP › additional file 2 (MEME logo)/HMA MEME logo .DOC]

**Sequence logo of the different motifs identified in the HMA gene family.**

 Motif 1

 Motif 2

 Motif 3

 Motif 4

 Motif 5

 Motif 6

 Motif 7

 Motif 8

Motif 9

 Motif 10

 Motif 11

 Motif 12

 Motif 13

 Motif 14

 Motif 15

 Motif 16

 Motif 17

 Motif 18

 Motif 19

 Motif 20
